# Positive mental attitude and depressive symptoms among medical students and residents: a cross-sectional study

**DOI:** 10.1371/journal.pone.0354032

**Published:** 2026-07-17

**Authors:** Xavier Sánchez, Pablo Carrera, María Daniela Cortez, Andrés Cruz, Isaac Merchán

**Affiliations:** 1 Community and Primary Care Research Group – Ecuador (CPCRG-E), Quito, Ecuador; 2 Centro de Investigación para la Salud en América Latina (CISeAL), Pontificia Universidad Católica del Ecuador (PUCE), Quito, Ecuador; 3 Postgrado de Medicina Familiar y Comunitaria, Pontificia Universidad Católica del Ecuador (PUCE), Quito, Ecuador; University of Toronto, CANADA

## Abstract

**Background:**

Depression is highly prevalent among medical trainees and negatively affects academic performance, empathy, and patient care. While healthy lifestyle behaviors are known to support mental health, the contribution of cognitive-emotional orientations, such as Positive Mental Attitude (PMA), remains underexplored. This study examined the associations between health behaviors, PMA, and depressive symptoms among medical students and residents in Ecuador.

**Methods:**

A cross-sectional survey was conducted in 2025 among 700 medical students and postgraduate residents at a private university. Participants completed the Patient Health Questionnaire-9 (PHQ-9) and the Health Behavior Inventory (HBI). Logistic regression models estimated associations between total HBI scores, its four subscales, and screening positive for clinically significant depressive symptoms (PHQ-9 ≥ 10), adjusting for sociodemographic and academic variables.

**Results:**

Nearly half of participants screened positive on the PHQ-9 (score ≥10) (47.7%). Higher total HBI scores were associated with lower odds of depressive symptoms (AOR = 0.94, 95% CI: 0.93–0.95). When HBI subscales were analyzed simultaneously, only PMA remained independently associated with lower odds of depressive symptoms (AOR = 0.79, 95% CI: 0.74–0.84). Female gender, younger age, and the presence of comorbidities were also associated with higher odds of depressive symptoms.

**Conclusions:**

PMA emerged as the behavioral domain most strongly associated with lower odds of clinically significant depressive symptoms in medical trainees, after adjustment for other lifestyle domains. These findings underscore the relevance of cognitive-emotional orientations in behavioral medicine and support the inclusion of positive psychology approaches in medical education to promote well-being.

## Introduction

Depression is one of the leading causes of disability worldwide and a major contributor to the global burden of disease [1]. Medical students and residents are particularly vulnerable, with prevalence rates of depressive symptoms consistently exceeding those of the general population [2,3]. This elevated risk has been linked to academic overload, long working hours, emotional demands of clinical care, and lack of adequate support structures [4, 5]. Beyond individual well-being, depressive symptoms negatively affect academic performance, empathy, and patient care [4,5], underscoring its relevance for public health and medical education.

While most research has focused on identifying risk factors for psychopathology, recent conceptual advances highlight the need to also investigate protective factors that sustain mental health. The World Health Organization conceptualizes mental health not merely as the absence of illness, but as a state of well-being encompassing functional capacity, resilience, and social contribution [6]. The dual-factor model distinguishes mental illness from positive mental health [7]. Evidence suggests that positive mental health (PMH)—encompassing optimism, self-acceptance, purpose in life, and positive relationships—independently predicts adaptive functioning, resilience, and remission from psychiatric disorders [8–10].

Within the framework of behavioral medicine, lifestyle practices such as physical activity, balanced diet, preventive care, and stress management are consistently associated with improved psychological outcomes [11]. Emerging evidence suggests that cognitive–emotional orientations represent an additional and distinct pathway through which health behaviors may be linked to mental and physical health outcomes. Optimism, for example, predicts lower incidence of coronary heart disease and reduced all-cause mortality [12–14], while positive affect has been linked to lower levels of inflammatory markers and stress-related physiological responses [15]. Within this perspective, Positive Mental Attitude (PMA) is especially relevant. Closely aligned with constructs such as optimism, resilience, and cognitive reappraisal, PMA reflects a tendency to sustain constructive emotional orientations in the face of adversity [16]. A growing body of evidence indicates that positive attitudes mitigate psychological distress and promote adaptive coping and long-term health benefits, as highlighted in recent integrative reviews [17]. However, little is known about how such orientations operate in medical trainees, a group uniquely exposed to chronic stressors and at elevated risk of mental health problems.

To address this gap, we examined the relationship between health behaviors and depressive symptoms in a large sample of medical students and postgraduate residents from a Latin American institution. Given the sustained exposure of medical trainees to academic, emotional, and clinical stressors, cognitive–emotional orientations such as PMA may play a particularly important role in shaping psychological responses to stress. Unlike other lifestyle domains, PMA reflects internal coping and appraisal processes, which may directly influence vulnerability to depressive symptoms in high-demand training environments. Using the Health Behavior Inventory (HBI), we investigated whether overall lifestyle patterns and specific behavioral domains, including PMA, were associated with depressive symptoms measured by the Patient Health Questionnaire-9 (PHQ-9). We hypothesized that while general healthy behaviors would be associated with lower odds of depressive symptoms, PMA would emerge as the most influential factor associated with psychological well-being in this population.

Understanding depressive symptoms among medical students and residents is particularly important given their dual role as learners and future healthcare providers. High psychological distress during training has been associated not only with impaired academic performance and professional development, but also with long-term consequences for workforce sustainability and patient care quality [4,18]. Identifying behavioral and cognitive–emotional correlates of depressive symptoms may therefore inform early, scalable strategies to support well-being during medical training.

## Methods

### Study design and setting

We conducted a cross-sectional study among undergraduate medical students and postgraduate residents at a private university in Ecuador, recognized nationally for medical education and specialty training. Data were collected from February 13 to March 31, 2025, during a standard academic term, in order to capture typical mental health and behavioral patterns while avoiding examination periods.

### Participants

The eligible population included all medical students (1,280) and residents (1,122) enrolled in 2025. The target sample size was calculated based on the total eligible population of undergraduate students and postgraduate residents enrolled in 2025. Assuming a 95% confidence level, a 5% margin of error, and a conservative prevalence estimate of 50% (to maximize variance in the absence of prior data), the finite population correction formula was applied, yielding a minimum required sample size of 287 participants.

Although this threshold was established to ensure adequate statistical precision for the primary outcome (clinically significant depressive symptoms, PHQ-9 ≥ 10), recruitment invitations were sent to the entire eligible population (census approach). A total of 700 participants (393 undergraduate students and 307 residents) completed the survey, exceeding the minimum required sample size and thereby increasing the precision of the estimates. Inclusion criteria were current enrollment and provision of informed consent.

Ethical approval was obtained from the PUCE Human Research Ethics Committee (approval number: EO-075–2024). The study was conducted in accordance with the principles of the Declaration of Helsinki. All participants provided informed consent electronically prior to participation. The online survey included an information sheet detailing the study objectives, procedures, risks, and confidentiality, and participants were required to indicate their consent by selecting an agreement option before accessing the questionnaire.

### Measures

#### Depressive symptoms.

Depressive symptoms were assessed with the Patient Health Questionnaire-9 (PHQ-9), a validated nine-item scale measuring symptom frequency over the prior two weeks (score range 0–27). A threshold of ≥10 was applied to define screening positive for clinically significant depressive symptoms, consistent with established sensitivity and specificity [19]. The Spanish version used has demonstrated strong psychometric properties in Ecuadorian populations [20,21]. Internal consistency in this sample was excellent (Cronbach’s α = 0.93).

### Health behaviors

The Health Behavior Inventory (HBI) [22,23] was used to assess health behaviors across four subscales: Healthy Eating Habits (EH), Preventive Behaviors (PB), Positive Mental Attitude (PMA), and Health Practices (HP). Items are rated on a five-point Likert scale (1 = almost never to 5 = almost always). The total HBI score is calculated as the sum of all 24 items, yielding a range from 24 to 120, with higher scores indicating healthier behaviors. Each of the four subscales consists of 6 items, with subscale scores ranging from 6 to 30. Example items include behaviors related to dietary patterns, preventive practices, and cognitive–emotional regulation (e.g., *“I avoid situations that depress me,” “I try to avoid stress and tensions,”* and *“I think positive”*). The Positive Mental Attitude (PMA) subscale captures cognitive and behavioral strategies related to emotional regulation. Although conceptually distinct from the clinical symptoms assessed by the PHQ-9, PMA reflects cognitive–emotional orientations such as optimism and self-regulation, which may be closely related to affective states. As such, some degree of conceptual overlap between PMA and depressive symptomatology cannot be excluded and should be considered when interpreting associations. A Spanish adaptation of the HBI, pilot-tested for linguistic clarity and suitability for Ecuadorian medical trainees, was used. The pilot included 15 residents, who provided feedback on item comprehension and survey flow; no substantive modifications were required. However, formal psychometric validation (e.g., factor structure, convergent validity, test–retest reliability) was not conducted in this study, which should be considered when interpreting the findings. Reliability in this sample was excellent (Cronbach’s α = 0.91).

### Covariates

Several sociodemographic and training-related variables were collected to contextualize outcomes. Sociodemographic variables included age (years), gender (male/female), marital status (single/married/divorced/stable union), children (yes/no), religious practice (yes/no), comorbidities (yes/no), living arrangement (alone/with family/with friends), and financial dependents (yes/no). Study hours and night shifts were reported as weekly averages. Night shifts applied primarily to postgraduate residents; undergraduate students typically reported none. Employment referred to paid work outside formal residency training. Alcohol use was assessed as a binary variable based on self-reported current consumption (yes/no). Tobacco use was assessed as a binary variable (“Do you currently use tobacco?” including cigarettes or cigars).

### Data collection

A secure online survey was distributed through institutional mailing lists. Unique access links ensured single responses, and weekly reminders were sent across four weeks. Data were collected anonymously and stored on a password-protected server. Only fully completed questionnaires were included in the analysis; partially completed surveys were excluded prior to analysis.

### Statistical analysis

Statistical analyses were conducted using R version 4.3.2. Descriptive statistics were used to summarize the characteristics of the sample. Categorical variables were presented as frequencies and percentages, while continuous variables were summarized using medians and interquartile ranges or means and standard deviations, as appropriate according to their distribution. Differences between participants with and without clinically significant depressive symptoms (PHQ-9 ≥ 10) were assessed using chi-square tests or Wilcoxon rank-sum tests, as appropriate.

Univariate logistic regression analyses were performed to examine the association between each independent variable and screening positive for clinically significant depressive symptoms (PHQ-9 ≥ 10). Variables with p ≤ 0.10 in univariate analyses, together with variables considered clinically relevant, were included in multivariable models. Additionally, models adjusted only for age and sex were fitted because these variables are well-established correlates of both depressive symptoms and health behaviors. These intermediate models were used to assess the robustness of the observed associations before inclusion of the full set of covariates.

Two multivariable models were then fitted. Model A was estimated using mixed-effects logistic regression including the total HBI score and selected covariates, with a random intercept for academic program (undergraduate vs postgraduate). This approach was used to account for potential clustering of participants within training programs, which may differ in workload, academic demands, and clinical responsibilities, potentially influencing mental health outcomes.

Model B included the four HBI subscales simultaneously, together with selected covariates, in order to assess their independent associations with depressive symptoms. The same mixed-effects structure was initially specified; however, the model showed singularity, indicating insufficient variability at the random-effect level to support reliable estimation of the mixed-effects structure. As a result, the inclusion of a random intercept was not statistically justified, and the final Model B was therefore estimated using fixed-effects logistic regression to ensure model stability and valid inference.

Model selection was guided by the Akaike Information Criterion (AIC). Odds ratios (ORs) and 95% confidence intervals (CIs) were reported, and statistical significance was set at p < 0.05 using two-tailed tests. Model assumptions were evaluated, including assessment of multicollinearity, model fit, and convergence, and no violations that would compromise the validity of the analyses were identified. Given the hypothesis-driven nature of the analyses and the pre-specification of exposure variables, no adjustment for multiple comparisons was applied.

## Results

### Sample characteristics

A total of 700 participants were included, comprising 393 undergraduates (56.14%) and 307 postgraduate residents (43.86%). The median age was 24 years (IQR 21–33), and 67.43% were female. Most participants were single (78.57%), lived with family (81.00%), and reported no comorbidities (77.43%).

When stratified by depressive symptoms, participants with clinically significant depressive symptoms (PHQ-9 ≥ 10) were younger, more frequently female, and more likely to be undergraduate students compared to those without depressive symptoms. They also had a higher prevalence of comorbidities and were less likely to report night shifts. In addition, participants with depressive symptoms had significantly lower HBI total scores compared to those without depressive symptoms (median 68.00 vs. 77.00; p < 0.001). Other sociodemographic and behavioral characteristics showed no consistent differences between groups (Table 1).

**Table 1:** Characteristics of participants according to depressive symptoms (PHQ-9 < 10 vs ≥ 10).

Variable	Total	PHQ-9 < 10	PHQ-9 ≥ 10	*p* value
**Age** (years), **median** (IQR)	24.00 (21.00–33.00)	27.50 (21.00–33.00)	23.00 (20.00–31.00)	<0.001
**Gender**				
Female	472 (67.43%)	225 (61.48%)	247 (73.95%)	0.001
Male	228 (32.57%)	141 (38.52%)	87 (26.05%)	
**Marital status**				
Divorced	19 (2.71%)	10 (2.73%)	9 (2.69%)	0.072
Married	106 (15.14%)	64 (17.49%)	42 (12.57%)	
Single	550 (78.57%)	284 (77.60%)	266 (79.64%)	
Stable union	25 (3.57%)	8 (2.19%)	17 (5.09%)	
**Program**				
Undergraduate	393 (56.14%)	178 (48.63%)	215 (64.37%)	<0.001
Postgraduate	307 (43.86%)	188 (51.37%)	119 (35.63%)	
**Religious practice**				
No	86 (12.29%)	38 (10.38%)	48 (14.37%)	0.136
Yes	614 (87.71%)	328 (89.62%)	286 (85.63%)	
**Living arrangement**				
Alone	110 (15.71%)	55 (15.03%)	55 (16.47%)	0.121
Family	567 (81.00%)	301 (82.24%)	266 (79.64%)	
Friend	15 (2.14%)	9 (2.46%)	6 (1.80%)	
Partner	8 (1.14%)	1 (0.27%)	7 (2.10%)	
**Children**				
No	562 (80.29%)	285 (77.87%)	277 (82.93%)	0.112
Yes	138 (19.71%)	81 (22.13%)	57 (17.07%)	
**Financial dependents**				
No	522 (74.57%)	270 (73.77%)	252 (75.45%)	0.673
Yes	178 (25.43%)	96 (26.23%)	82 (24.55%)	
**Alcohol use**				
Yes	466 (66.57%)	248 (67.76%)	218 (65.27%)	0.537
No	234 (33.43%)	118 (32.24%)	116 (34.73%)	
**Tobacco use**				
Yes	85 (12.14%)	36 (9.84%)	49 (14.67%)	0.066
No	615 (87.86%)	330 (90.16%)	285 (85.33%)	
**Presence of comorbidities**				
No	542 (77.43%)	300 (81.97%)	242 (72.46%)	0.004
Yes	158 (22.57%)	66 (18.03%)	92 (27.54%)	
**Hours of study**, **median** (IQR)	40.00 (11.75–70.00)	50.00 (10.00–70.00)	34.00 (12.00–70.00)	0.571
**Financial support**				
Self-pay	533 (76.14%)	284 (77.60%)	249 (74.55%)	0.392
Scholarship	167 (23.86%)	82 (22.40%)	85 (25.45%)	
**Night shifts**				
Yes	361 (51.57%)	207 (56.56%)	154 (46.11%)	0.007
No	339 (48.43%)	159 (43.44%)	180 (53.89%)	
**Work**				
No	580 (82.86%)	305 (83.33%)	275 (82.34%)	0.803
Yes	120 (17.14%)	61 (16.67%)	59 (17.66%)	
Health Behavior Inventory **(HBI)** total score**, median** (IQR)	73.00 (64.00–81.00)	77.00 (69.00–84.00)	68.00 (60.00–77.00)	<0.001

**HBI:** Health Behavior Inventory, **IQR:** Interquartile range

Among participants reporting comorbidities, mental health conditions were the most frequent (43.67%), followed by endocrine-metabolic (18.98%) and cardiovascular conditions (12.66%).

### Prevalence of healthy behaviors and clinically significant depressive symptoms

Table 2 presents the distribution of HBI subscale scores according to depressive symptom status. Participants with PHQ-9 ≥ 10 had significantly lower scores across all subscales compared to those without depressive symptoms (all p < 0.001).

**Table 2:** Distribution of HBI subscale scores according to depressive symptom status.

Subscale	Total	PHQ-9 < 10	PHQ-9 ≥ 10	*p* value
**Healthy Eating Habits (EH),** median **(IQR)**	18.00 (15.00–21.00)	19.00 (16.00–21.75)	17.00 (14.00–20.00)	<0.001
**Preventive Behaviors (PB),** median **(IQR)**	18.00 (15.00–21.00)	19.00 (16.00–21.75)	17.00 (14.00–20.00)	<0.001
**Positive Mental Attitude (PMA),** median **(IQR)**	19.00 (17.00–22.00)	21.00 (18.00–23.00)	18.00 (15.00–20.00)	<0.001
**Health Practices (HP),** median **(IQR)**	17.00 (15.00–20.00)	18.00 (16.00–20.00)	16.00 (14.00–19.00)	<0.001

**IQR:** Interquartile range

**Fig 1:**
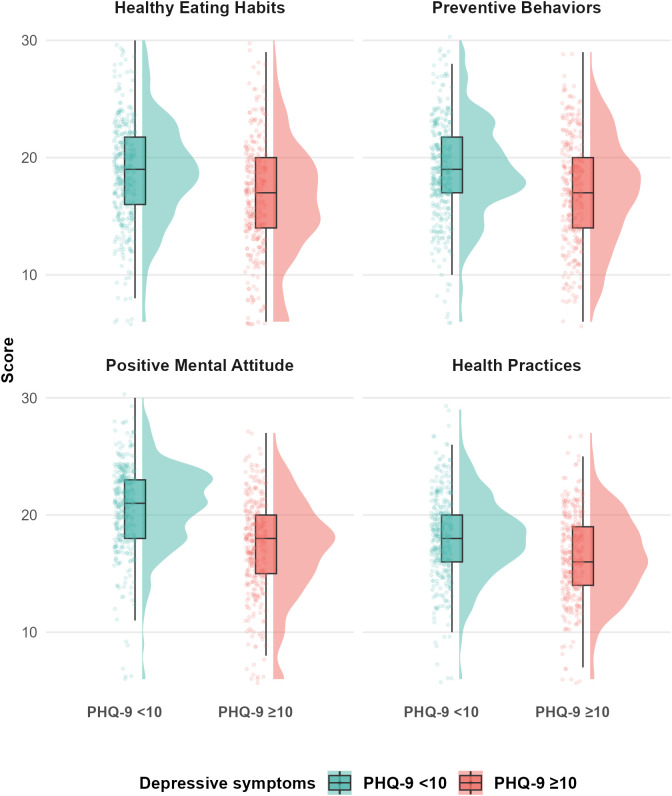
Distribution of Health Behavior Inventory (HBI) subscale scores according to depressive symptom status (PHQ-9 < 10 vs PHQ-9 ≥ 10). Raincloud plots display the distribution of scores for each subscale (Healthy Eating Habits, Preventive Behaviors, Positive Mental Attitude, and Health Practices), including density estimates, individual observations, and boxplots summarizing the median and interquartile range. Corresponding statistical comparisons between groups are reported in Table 2.

Overall, 334 participants (47.70%, 95% CI: 44.01–51.41) screened positive for depressive symptoms (PHQ-9 ≥ 10). Participants with PHQ-9 ≥ 10 had significantly lower total HBI scores compared to those without depressive symptoms (Median = 68.00 vs. 77.00; IQR = 60–77 vs. 69–84; *p* < 0.001). When analyzing the four HBI subscales individually, participants with PHQ-9 ≥ 10 also reported lower scores in Healthy Eating Habits (*p* < 0.001), Preventive Behaviors (*p* < 0.001), Positive Mental Attitude (*p* < 0.001), and Health Practices (*p* < 0.001), based on Wilcoxon rank-sum tests (Fig 1).

### Exploratory analysis

All reported associations refer to clinically significant depressive symptoms as defined by a PHQ-9 score ≥10. Univariate analyses were performed as part of the model-building process (Table 3). Higher HBI scores were associated with lower odds of having a PHQ-9 ≥ 10 (OR = 0.95 per point, 95% CI: 0.94–0.96, p < 0.001). Each HBI subscale showed similar inverse associations: EH (OR = 0.90, 95% CI: 0.87–0.93), PB (OR = 0.92, 95% CI: 0.88–0.95), PMA (OR = 0.79, 95% CI: 0.75–0.82), and HP (OR = 0.87, 95% CI: 0.84–0.91), all p < 0.001. Younger age (<24 years; OR = 1.76, 95% CI: 1.31–2.38, p < 0.001), female gender (OR = 1.78, 95% CI: 1.29–2.46, p < 0.001), comorbidities (OR = 1.73, 95% CI: 1.21–2.48, p = 0.003), and undergraduate status (OR = 1.91, 95% CI: 1.41–2.59, p < 0.001) were also significantly associated. Night shifts were associated with reduced odds (OR = 0.66, 95% CI: 0.49–0.89, p = 0.006). Other variables were not significant.

**Table 3:** Univariate logistic regression: Associations of health behaviors, sociodemographic, academic, and clinical variables with depressive symptoms (PHQ-9 ≥ 10).

Variable	Category	OR	95% CI	*p* value
**HBI total score**	Per 1-point increase	0.95	0.94–0.96	**<0.001**
**Healthy Eating Habits**	Per 1-point increase	0.90	0.87–0.93	**<0.001**
**Preventive Behaviors**	Per 1-point increase	0.92	0.88–0.95	**<0.001**
**Positive Mental Attitude**	Per 1-point increase	0.79	0.75–0.82	**<0.001**
**Health Practices**	Per 1-point increase	0.87	0.84–0.91	**<0.001**
**Age group**	< 24 years vs ≥ 24 years	1.76	1.31–2.38	**<0.001**
**Alcohol use**	Yes vs No	0.89	0.65–1.22	0.486
**Children**	No vs Yes	1.38	0.95–2.02	0.093
**Comorbidity**	Yes vs No	1.73	1.21–2.48	**0.003**
**Dependents**	No vs Yes	1.09	0.78–1.54	0.611
**Financial support**	Self-pay vs Scholarship	0.85	0.60–1.20	0.345
**Gender**	Female vs Male	1.78	1.29–2.46	**<0.001**
**Hours of study**	≥ 40 hours vs < 40 hours	0.76	0.56–1.02	0.066
**Living arrangement**	Alone vs Accompanied	1.11	0.74–1.68	0.601
**Marital status** (ref: Single)	Divorced	2.36	0.70–8.37	0.172
	Married	0.73	0.27–1.98	0.528
	Stable union	1.04	0.41–2.66	0.932
**Night shifts**	Yes vs No	0.66	0.49–0.89	**0.006**
**Program**	Undergraduate vs Postgraduate	1.91	1.41–2.59	**<0.001**
**Religious practice**	No vs Yes	1.45	0.92–2.29	0.110
**Tobacco use**	Yes vs No	1.58	1.00–2.51	0.052
**Work**	Yes vs No	1.07	0.72–1.59	0.726

**OR:** Odds ratio, **CI:** Confidence Interval, **HBI:** Health Behavior Inventory

### Multivariate analysis

Table 4 shows the results of Model A. Higher HBI scores remained independently associated with lower odds of depressive symptoms (AOR = 0.94, 95% CI: 0.93–0.95, p < 0.001), indicating that each one-point increase in the HBI score was associated with a 6% reduction in the odds of screening positive for depressive symptoms. Female gender (AOR = 1.76, 95% CI: 1.21–2.55, p = 0.003) and the presence of comorbidities (AOR = 1.90, 95% CI: 1.26–2.84, p = 0.002) were also independently associated.

**Table 4:** Multivariate logistic regression (Model A): associations of total health behavior inventory (HBI) score and covariates with clinically significant depressive symptoms (PHQ-9 ≥ 10).

Variable	Category	AOR	95% CI	*p* value
**HBI Score**	Per 1-point increase	0.94	0.93–0.95	**<0.001**
**Age group**	< 24 years vs ≥ 24 years	1.78	0.78–3.23	0.205
**Gender**	Female vs Male	1.76	1.21–2.55	**0.003**
**Comorbidity**	Yes vs No	1.90	1.26–2.84	**0.002**
**Night shifts**	Yes vs No	1.19	0.60–2.39	0.617
**Tobacco use**	Yes vs No	1.22	0.72–2.06	0.463
**Religious practice**	No vs Yes	1.46	0.87–2.44	0.150
**Hours of study**	≥ 40 hours vs < 40 hours	1.05	0.68–1.62	0.838
**Children**	No vs Yes	1.01	0.62–1.64	0.971

**AOR:** Adjusted odds ratio; **CI:** Confidence Interval. The reference category corresponds to the second group in each comparison.

Table 5 depicts the results of Model B. Only positive mental attitude (PMA) was independently associated with depressive symptoms (AOR = 0.79, 95% CI: 0.74–0.84, p < 0.001), indicating that each one-point increase in the PMA score was associated with a 21% reduction in the odds of screening positive for depressive symptoms. In contrast, EH, PB, and HP were not independently associated after adjustment. Younger age (<24 years; AOR = 1.92, 95% CI: 1.05–3.56, p = 0.036), female gender (AOR = 1.74, 95% CI: 1.18–2.58, p = 0.005), and the presence of comorbidities (AOR = 1.67, 95% CI: 1.10–2.55, p = 0.017) were also significantly associated in this model.

**Table 5:** Multivariate logistic regression (Model B): associations of health behavior inventory (subscales) and covariates with clinically significant depressive symptoms (PHQ-9 ≥ 10).

Variable	Category	AOR	95% CI	*p* value
**Healthy Eating Habits**	Per 1-point increase	0.96	0.92–1.01	0.149
**Preventive Behaviors**	Per 1-point increase	1.05	0.99–1.11	0.120
**Positive Mental Attitude**	Per 1-point increase	0.79	0.74–0.84	**<0.001**
**Health Practices**	Per 1-point increase	0.96	0.90–1.02	0.206
**Age group**	< 24 years vs ≥ 24 years	1.92	1.05–3.56	**0.036**
**Gender**	Female vs Male	1.74	1.18–2.58	**0.005**
**Comorbidity**	Yes vs No	1.67	1.10–2.55	**0.017**
**Night shifts**	Yes vs No	0.92	0.50–1.71	0.783
**Tobacco use**	Yes vs No	1.25	0.73–2.15	0.423
**Religious practice**	No vs Yes	1.53	0.90–2.62	0.119
**Hours of study**	≥ 40 hours vs < 40 hours	0.99	0.67–1.48	0.969
**Children**	No vs Yes	0.97	0.59–1.58	0.893

**AOR:** Adjusted odds ratio; **CI:** Confidence Interval. The reference category corresponds to the second group in each comparison.

Additional models adjusted only for age and sex showed results broadly consistent with those of the fully adjusted models (S1 Table). In particular, the inverse association between total HBI score and depressive symptoms, as well as the association with the positive mental attitude subscale, remained stable across model specifications.

## Discussion

In this cross-sectional study of undergraduate and postgraduate medical trainees, we observed a high prevalence of clinically significant depressive symptoms. Higher total HBI scores were consistently associated with lower odds of depressive symptoms. Among the HBI subscales, positive mental attitude demonstrated the strongest and most consistent independent association observed, whereas other subscales were not significant after adjustment. The independent association between PMA and depressive symptoms that remained after adjustment for other HBI domains and covariates may partly reflect conceptual overlap between PMA and affective states, as well as a bidirectional relationship between cognitive–emotional regulation and depressive symptoms. At the same time, it is also plausible that higher levels of PMA may exert a protective effect by promoting adaptive coping strategies, emotional resilience, and more effective responses to stress, thereby reducing vulnerability to depressive symptoms. Beyond health behaviors, female gender and the presence of comorbidities were also independently associated with depressive symptoms.

Another notable finding was the high prevalence of clinically significant depressive symptoms in our sample. Nearly half of medical students and residents had PHQ-9 scores ≥10 (47.7%), a prevalence substantially higher than that reported in international meta-analyses. Global estimates suggest that approximately 27% of medical students and 29% of residents screen positive for clinically significant depressive symptoms [2,5]. More recent reviews indicate that between one-quarter and one-third of trainees are affected worldwide [24], underscoring that our findings exceed global averages. Evidence from Latin America also points to an elevated burden of depressive symptoms among healthcare workers. During the COVID-19 pandemic, pooled prevalence estimates of depression among healthcare workers across 11 countries ranged from 14.7% to 22.0%, reinforcing a challenge that was evident during the crisis and appears to persist in the aftermath [25]. In addition, a study in Peru reported that approximately 30% of medical students experienced moderate depressive symptoms during the pandemic’s second wave [26], while a study in Mexico identified a prevalence of 49% among medical students [27]. Taken together, these findings suggest that the psychological burden in Latin American medical training settings may exceed global averages, possibly due to systemic educational stressors, cultural stigma around mental health, and resource constraints. Given the consequences of depressive symptoms for empathy, learning, and patient care [4,18], the observed rates underscore an urgent need for regionally tailored support measures and interventions.

Beyond overall prevalence, these findings suggest that certain subgroups may be particularly vulnerable. Specifically, younger participants, females, and those reporting comorbidities consistently showed higher odds of depressive symptoms, indicating a potential clustering of risk factors. Although subgroup-specific analyses were beyond the scope of this study, these patterns highlight the importance of targeted screening and support strategies for higher-risk groups within medical training environments.

Our findings suggest that not all health behavior domains contribute equally to the observed association with depressive symptoms. Although healthier lifestyle patterns overall were associated with lower odds of depressive symptoms, only PMA remained independently associated after adjustment for the other HBI domains and covariates. This finding may indicate that cognitive–emotional processes are particularly relevant in shaping psychological well-being among medical trainees, a population exposed to persistent academic and clinical stressors. While evidence from randomized trials and systematic reviews supports the role of lifestyle interventions, including nutrition, physical activity, sleep, and stress management, in promoting mental health [28–33], our results suggest that cognitive–emotional orientations such as PMA may represent an especially salient component within broader behavioral medicine frameworks. Future studies should explore whether interventions aimed at strengthening adaptive coping, emotional regulation, and resilience can improve mental health outcomes in medical trainees.

Interestingly, performing night shifts was associated with lower odds of depressive symptoms in univariate analysis, a finding that contrasts with existing literature generally linking shift work to adverse mental health outcomes [33,34]. This result may reflect residual confounding, selection effects (e.g., more resilient individuals being more likely to engage in night shifts), contextual factors related to training structure, or the normalization and expectation of night shift work within medical training environments, and should therefore be interpreted with caution.

Beyond their theoretical relevance, these findings also have practical implications for medical training environments. Because PMA was the only HBI domain that remained independently associated with depressive symptoms after adjustment, cognitive–emotional regulation may represent a particularly relevant target for intervention in this population. These findings suggest that screening for cognitive–emotional orientations such as PMA may complement traditional lifestyle assessments in medical training settings. Low-cost interventions aimed at fostering adaptive cognitive framing, emotional regulation, and stress management could be integrated into student support services and residency wellness programs without requiring substantial structural changes. This recommendation is supported by prior evidence indicating that mindfulness-based and cognitive-behavioral interventions can improve stress, anxiety, and depressive symptoms in health-professions students and trainees, although the certainty of evidence varies across intervention types [35–38]. Interventions for medical students may prioritize academic workload management and peer support, whereas residency programs may benefit from duty-hour optimization and cognitive resilience training.

We recognize both the strengths and limitations of our study. Among its strengths, it relied on a large and heterogeneous sample that included both medical students and postgraduate residents, which increases the robustness of the results. The use of validated and widely employed instruments with excellent internal consistency also adds credibility to our findings. Moreover, this study contributes novel evidence from a Latin American medical training context and highlights the relative salience of cognitive–emotional health behaviors compared with traditional lifestyle domains when examined simultaneously. Importantly, by evaluating lifestyle behaviors and cognitive-emotional orientations within the same framework, we were able to demonstrate that PMA stands out as a salient behavioral factor of psychological well-being, adding new insights to the behavioral medicine literature.

At the same time, several limitations must be acknowledged. First, the cross-sectional design prevents the establishment of causal relationships and limits the ability to determine the directionality of the association between health behaviors, including PMA, and depressive symptoms. Additionally, the response rate may introduce potential non-response bias, as individuals with more severe depressive symptoms or poorer health behaviors may have been less likely to participate, potentially leading to an underestimation of the true prevalence and strength of associations. Furthermore, although a Spanish adaptation of the HBI was pilot-tested for clarity and showed good internal consistency, formal psychometric validation in this population was not conducted, which may limit the interpretability of the measured constructs. Although the study was conducted at a single institution, postgraduate residents were recruited from diverse health services and cities across the country, which may partially mitigate this limitation. Finally, because prior diagnoses of depression or other mental disorders were not used as exclusion criteria, it is possible that pre-existing conditions contributed to the observed prevalence and associations, potentially inflating estimates of depressive symptoms.

Although female sex was associated with higher odds of depressive symptoms, we did not perform sex-stratified analyses; future studies could explore potential differences in these associations across sex. In addition, some covariates (e.g., night shifts, paid work, and hours of study) may have different relevance depending on training level; however, models were adjusted for academic program and age to partially account for these differences. Furthermore, the predominance of female participants in the sample may have influenced the observed prevalence and associations. Moreover, the analysis did not stratify by training stage or curricular phase, which may further limit the interpretation of differences across academic levels. Finally, we are also aware that reliance on self-reported measures may introduce bias, and that unmeasured confounders cannot be excluded. Even so, the consistency of the observed associations, together with the integrated assessment of lifestyle and cognitive–emotional domains, supports the contribution of this study and points to clear directions for future multicenter and longitudinal research.

## Conclusion

This study shows that clinically significant depressive symptoms are highly prevalent among medical students and residents in this setting, underscoring the persistent psychological burden in this population. While healthier lifestyle behaviors overall were associated with lower odds of depressive symptoms, Positive Mental Attitude emerged as the only behavioral domain independently associated with depressive symptoms in multivariable models. These findings should be interpreted as associative rather than causal and may partly reflect both conceptual proximity and a bidirectional relationship between cognitive–emotional regulation and depressive symptoms. Nevertheless, they highlight the potential relevance of cognitive–emotional orientations within behavioral medicine frameworks and support further longitudinal and interventional research to clarify their role in promoting mental well-being among medical trainees.

## Supporting information

S1 Table Age- and sex-adjusted, and fully adjusted logistic regression models for the association between Health Behavior Inventory (HBI) (total score and subscales) and depressive symptoms. (PDF)

## References

[1] World Health Organization. Comprehensive Mental Health Action Plan 2013–2030. Geneva. 2021. https://www.who.int/publications/i/item/9789240031029

[2] Rotenstein LS, Ramos MA, Torre M, Segal JB, Peluso MJ, Guille C, et al. Prevalence of Depression, Depressive Symptoms, and Suicidal Ideation Among Medical Students. JAMA. 2016;316(21):2214. doi: 10.1001/jama.2016.17324 27923088 PMC5613659

[3] Puthran R, Zhang MWB, Tam WW, Ho RC. Prevalence of depression amongst medical students: a meta-analysis. Med Educ. 2016;50(4):456–68. doi: 10.1111/medu.12962 26995484

[4] Dyrbye LN, Thomas MR, Shanafelt TD. Medical student distress: causes, consequences, and proposed solutions. Mayo Clin Proc. 2005;80:1613–22. doi: 10.4065/80.12.1613 16342655

[5] Mata DA, Ramos MA, Bansal N, Khan R, Guille C, Di Angelantonio E, et al. Prevalence of Depression and Depressive Symptoms Among Resident Physicians. JAMA. 2015;314(22):2373. doi: 10.1001/jama.2015.15845 26647259 PMC4866499

[6] Herrman H, Saxena S, Moodie R. Promoting mental health: concepts, emerging evidence, practice. Geneva: World Health Organization. 2005. https://www.who.int/publications/i/item/9241562943

[7] Westerhof GJ, Keyes CLM. Mental Illness and Mental Health: The Two Continua Model Across the Lifespan. J Adult Dev. 2010;17(2):110–9. doi: 10.1007/s10804-009-9082-y 20502508 PMC2866965

[8] Keyes CLM, Dhingra SS, Simoes EJ. Change in level of positive mental health as a predictor of future risk of mental illness. Am J Public Health. 2010;100(12):2366–71. doi: 10.2105/AJPH.2010.192245 20966364 PMC2978199

[9] Lamers SMA, Westerhof GJ, Glas CAW, Bohlmeijer ET. The bidirectional relation between positive mental health and psychopathology in a longitudinal representative panel study. J Posit Psychol. 2015;10:553–60. doi: 10.1080/17439760.2015.1015156

[10] Schotanus-Dijkstra M, Keyes CLM, de Graaf R, Ten Have M. Recovery from mood and anxiety disorders: The influence of positive mental health. J Affect Disord. 2019;252:107–13. doi: 10.1016/j.jad.2019.04.051 30981053

[11] Walsh R. Lifestyle and mental health. American Psychologist. 2011;66:579–92. doi: 10.1037/a0021769 21244124

[12] Kubzansky LD, Sparrow D, Vokonas P, Kawachi I. Is the glass half empty or half full? A prospective study of optimism and coronary heart disease in the normative aging study. Psychosom Med. 2001;63(6):910–6. doi: 10.1097/00006842-200111000-00009 11719629

[13] Giltay EJ, Geleijnse JM, Zitman FG, Hoekstra T, Schouten EG. Dispositional optimism and all-cause and cardiovascular mortality in a prospective cohort of elderly dutch men and women. Arch Gen Psychiatry. 2004;61(11):1126–35. doi: 10.1001/archpsyc.61.11.1126 15520360

[14] Rozanski A, Bavishi C, Kubzansky LD, Cohen R. Association of Optimism With Cardiovascular Events and All-Cause Mortality. JAMA Netw Open. 2019;2:e1912200. doi: 10.1001/jamanetworkopen.2019.12200 PMC6777240 31560385

[15] Steptoe A, Dockray S, Wardle J. Positive affect and psychobiological processes relevant to health. J Pers. 2009;77:1747–76. doi: 10.1111/j.1467-6494.2009.00599.x 19796062 PMC2787693

[16] Scheier MF, Carver CS. Dispositional optimism and physical health: A long look back, a quick look forward. Am Psychol. 2018;73(9):1082–94. doi: 10.1037/amp0000384 30525784 PMC6309621

[17] Margraf J, Teismann T, Brailovskaia J. Predictive power of positive mental health: A scoping review. J Happiness Stud. 2024;25:81. doi: 10.1007/s10902-024-00788-x

[18] Li C, Wu H. Association between medical students’ psychological distress and intention to practice. BMC Med Educ. 2026;26(1):899. doi: 10.1186/s12909-026-09257-w 42015127 PMC13235139

[19] Kroenke K, Spitzer RL, Williams JBW. The PHQ-9. J Gen Intern Med. 2001;16:606–13. doi: 10.1046/j.1525-1497.2001.016009606.x 11556941 PMC1495268

[20] López-Guerra VM, López-Núñez C, Vaca-Gallegos SL, Torres-Carrión PV. Psychometric Properties and Factor Structure of the Patient Health Questionnaire-9 as a Screening Tool for Depression Among Ecuadorian College Students. Front Psychol. 2022;13:813894. doi: 10.3389/fpsyg.2022.813894 35572338 PMC9105228

[21] Quiñonez-Freire C, Vara MD, Tomás JM, Baños RM. Psychometric properties of the Spanish version of the Patient Health Questionnaire-9 in users of the Ecuadorian public health care system. Rev Latinoam Psicol. 2022;53. doi: 10.14349/rlp.2021.v53.23

[22] Juczyński Z. Narzędzia pomiaru w promocji i psychologii zdrowia. Pracownia Testów Psychologicznych Polskiego Towarzystwa Psychologicznego, editor. Warszawa: Pracownia Testów Psychologicznych Polskiego Towarzystwa Psychologicznego. 2001.

[23] Kraśnicka J, Krajewska-Kułak E, Klimaszewska K, Cybulski M, Guzowski A, Lewko J, et al. The impact of parents’ health behaviours on their preferences regarding vaccinations in Bialystok, Poland. BMC Pediatr. 2020;20(1):354. doi: 10.1186/s12887-020-02235-1 32711498 PMC7381861

[24] Quek TT-C, Tam WW-S, Tran BX, Zhang M, Zhang Z, Ho CS-H, et al. The Global Prevalence of Anxiety Among Medical Students: A Meta-Analysis. Int J Environ Res Public Health. 2019;16(15):2735. doi: 10.3390/ijerph16152735 31370266 PMC6696211

[25] Pan American Health Organization. The COVID-19 health care workers study (HEROES): Regional report from the Americas. 2022. https://iris.paho.org/handle/10665.2/55972

[26] Valladares-Garrido D, Quiroga-Castañeda PP, Berrios-Villegas I, Zila-Velasque JP, Anchay-Zuloeta C, Chumán-Sánchez M, et al. Depression, anxiety, and stress in medical students in Peru: a cross-sectional study. Front Psychiatry. 2023;14:1268872. doi: 10.3389/fpsyt.2023.1268872 38090694 PMC10715266

[27] Robles-Rivera K, Limón-Rojas AE, Wakida-Kuzunoki GH, Moreno-Altamirano L, Vázquez-Rivera M, Romero-Romero E. Factors associated with depression, anxiety, and stress in Mexican medical students: a cross-sectional study. Current Psychology. 2025;44:9252–64. doi: 10.1007/s12144-025-07742-x

[28] Jacka FN, O’Neil A, Opie R, Itsiopoulos C, Cotton S, Mohebbi M, et al. A randomised controlled trial of dietary improvement for adults with major depression (the “SMILES” trial). BMC Med. 2017;15(1):23. doi: 10.1186/s12916-017-0791-y 28137247 PMC5282719

[29] Schuch FB, Vancampfort D, Firth J, Rosenbaum S, Ward PB, Silva ES, et al. Physical Activity and Incident Depression: A Meta-Analysis of Prospective Cohort Studies. Am J Psychiatry. 2018;175(7):631–48. doi: 10.1176/appi.ajp.2018.17111194 29690792

[30] Freeman D, Sheaves B, Goodwin GM, Yu L-M, Nickless A, Harrison PJ, et al. The effects of improving sleep on mental health (OASIS): a randomised controlled trial with mediation analysis. Lancet Psychiatry. 2017;4(10):749–58. doi: 10.1016/S2215-0366(17)30328-0 28888927 PMC5614772

[31] Wong VW-H, Ho FY-Y, Shi N-K, Sarris J, Chung K-F, Yeung W-F. Lifestyle medicine for depression: A meta-analysis of randomized controlled trials. J Affect Disord. 2021;284:203–16. doi: 10.1016/j.jad.2021.02.012 33609955

[32] Slavin SJ, Schindler DL, Chibnall JT. Medical Student Mental Health 3.0. Academic Medicine. 2014;89:573–7. doi: 10.1097/ACM.0000000000000166 24556765 PMC4885556

[33] Xu M, Yin X, Gong Y. Lifestyle Factors in the Association of Shift Work and Depression and Anxiety. JAMA Netw Open. 2023;6(8):e2328798. doi: 10.1001/jamanetworkopen.2023.28798 37578795 PMC10425829

[34] Lee A, Myung SK, Cho JJ, Jung YJ, Yoon JL, Kim MY. Night Shift Work and Risk of Depression: Meta-analysis of Observational Studies. J Korean Med Sci. 2017;32(7):1091–6. doi: 10.3346/jkms.2017.32.7.1091 28581264 PMC5461311

[35] da Silva CCG, Bolognani CV, Amorim FF, Imoto AM. Effectiveness of training programs based on mindfulness in reducing psychological distress and promoting well-being in medical students: a systematic review and meta-analysis. Syst Rev. 2023;12(1):79. doi: 10.1186/s13643-023-02244-y 37147732 PMC10160720

[36] Lu C-P, Dijk SW, Pandit A, Kranenburg L, Luik AI, Hunink MGM. The effect of mindfulness-based interventions on reducing stress in future health professionals: A systematic review and meta-analysis of randomized controlled trials. Appl Psychol Health Well Being. 2024;16(2):765–92. doi: 10.1111/aphw.12472 37527644

[37] Melnyk BM, Kelly SA, Stephens J, Dhakal K, McGovern C, Tucker S, et al. Interventions to Improve Mental Health, Well-Being, Physical Health, and Lifestyle Behaviors in Physicians and Nurses: A Systematic Review. Am J Health Promot. 2020;34(8):929–41. doi: 10.1177/0890117120920451 32338522 PMC8982669

[38] Kunzler AM, Helmreich I, Chmitorz A, König J, Binder H, Wessa M, et al. Psychological interventions to foster resilience in healthcare professionals. Cochrane Database Syst Rev. 2020;7(7):CD012527. doi: 10.1002/14651858.CD012527.pub2 32627860 PMC8121081

