# Supplementary material for: Positive mental attitude and depressive symptoms among medical students and residents: a cross-sectional study
Source: PLoS One. 2026 Jul 17;21(7):e0354032. doi: 10.1371/journal.pone.0354032 (PMC13378974; doi:10.1371/journal.pone.0354032)
Supplement: S1 Table — (PDF) [file pone.0354032.s001.pdf]

S1 Table. Age- and sex-adjusted, and fully adjusted logistic regression models for the association between Health Behavior Inventory (HBI) (total score and subscales) and depressive symptoms

A. Association between total HBI score and depressive symptoms

Variable	Model	OR	95% CI	p-value
HBI total score	Age- and sex-adjusted	0.94	0.93–0.95	<0.001
HBI total score	Fully adjusted	0.94	0.93–0.95	<0.001
Age <24	Age- and sex-adjusted	2.23	1.59–3.14	<0.001
Age <24	Fully adjusted	1.78	0.78–3.23	0.205
Female	Age- and sex-adjusted	1.79	1.26–2.57	0.001
Female	Fully adjusted	1.76	1.21–2.55	0.003

B. Association between HBI subscales and depressive symptoms

Variable	Model	OR	95% CI	p-value
Healthy Eating Habits	Age- and sex-adjusted	0.97	0.92–1.02	0.235
Healthy Eating Habits	Fully adjusted	0.96	0.92–1.01	0.149
Preventive Behaviors	Age- and sex-adjusted	1.05	1.00–1.11	0.072
Preventive Behaviors	Fully adjusted	1.05	0.99–1.11	0.120
Positive Mental Attitude	Age- and sex-adjusted	0.79	0.74–0.84	<0.001
Positive Mental Attitude	Fully adjusted	0.79	0.74–0.84	<0.001
Health Practices	Age- and sex-adjusted	0.95	0.89–1.01	0.080
Health Practices	Fully adjusted	0.96	0.90–1.02	0.206
Age <24	Age- and sex-adjusted	1.99	1.38–2.89	<0.001
Age <24	Fully adjusted	1.92	1.05–3.56	0.036
Female	Age- and sex-adjusted	1.74	1.20–2.54	0.004
Female	Fully adjusted	1.74	1.18–2.58	0.005

OR: Odds ratio; CI: Confidence interval. Age- and sex-adjusted models include adjustment for age and sex. Fully adjusted models include all covariates presented in the main models (Tables 4 and 5).
