# Supplementary material for: Positive mental attitude and depressive symptoms among medical students and residents: a cross-sectional study
Source: PLoS One. 2026 Jul 17;21(7):e0354032. doi: 10.1371/journal.pone.0354032 (PMC13378974; doi:10.1371/journal.pone.0354032)
Supplement: Attachment — Submitted filename: Response to reviewers.docx [file pone.0354032.s003.docx]

**Point-by-point respond letter**

**Editor’s comments**

***1. Please ensure that your manuscript meets PLOS ONE's style requirements, including those for file naming. The PLOS ONE style templates can be found at***

We thank the editor for this reminder. We confirm that the manuscript has been revised to comply with PLOS ONE’s style requirements, including file naming and formatting guidelines.

***2. Please provide details regarding participant consent. In the ethics statement in the Methods and online submission information, please ensure that you have specified (1) whether consent was informed and (2) what type you obtained (for instance, written or verbal, and if verbal, how it was documented and witnessed). If your study included minors, state whether you obtained consent from parents or guardians. If the need for consent was waived by the ethics committee, please include this information.***

We thank the editor for this clarification. We have revised the ethics statement to specify that informed consent was obtained electronically. Participants were provided with an online information sheet and indicated their consent by selecting an agreement option before accessing the survey.

***If you are reporting a retrospective study of medical records or archived samples, please ensure that you have discussed whether all data were fully anonymized before you accessed them and/or whether the IRB or ethics committee waived the requirement for informed consent. If patients provided informed written consent to have data from their medical records used in research, please include this information.***

We thank the editor for this clarification. This requirement is not applicable to our study, as it did not involve retrospective medical records or archived samples. Instead, data were collected prospectively through an anonymous online survey, and all participants provided informed electronic consent prior to participation.

***3. We note that you have indicated that there are restrictions to data sharing for this study. For studies involving human research participant data or other sensitive data, we encourage authors to share de-identified or anonymized data. However, when data cannot be publicly shared for ethical reasons, we allow authors to make their data sets available upon request. For information on unacceptable data access restrictions, please see http://journals.plos.org/plosone/s/data-availability#loc-unacceptable-data-access-restrictions.***

***Before we proceed with your manuscript, please address the following prompts:***

***a) If there are ethical or legal restrictions on sharing a de-identified data set, please explain them in detail (e.g., data contain potentially identifying or sensitive patient information, data are owned by a third-party organization, etc.) and who has imposed them (e.g., a Research Ethics Committee or Institutional Review Board, etc.). Please also provide contact information for a data access committee, ethics committee, or other institutional body to which data requests may be sent.***

***b) If there are no restrictions, please upload the minimal anonymized data set necessary to replicate your study findings to a stable, public repository and provide us with the relevant URLs, DOIs, or accession numbers. Please see http://www.bmj.com/content/340/bmj.c181.long for guidelines on how to de-identify and prepare clinical data for publication. For a list of recommended repositories, please see https://journals.plos.org/plosone/s/recommended-repositories. You also have the option of uploading the data as Supporting Information files, but we would recommend depositing data directly to a data repository if possible.***

***Please update your Data Availability statement in the submission form accordingly.***

We thank the editor for this clarification. There are no ethical or legal restrictions on data sharing. The dataset has been fully anonymized and is publicly available in a stable repository. The minimal dataset required to replicate the study findings has been deposited in Mendeley Data: [*https://data.mendeley.com/preview/vx7fdzkjj5*](https://data.mendeley.com/preview/vx7fdzkjj5). *Sánchez, Xavier (2026), “Dataset on Depression Symptoms, Health Behaviors, and Academic Characteristics among Medical Students and Resident Physicians in Ecuador”, Mendeley Data, V2, doi: 10.17632/vx7fdzkjj5.2*

***4. In the online submission form, you indicated that de-identified data may be made available upon reasonable request to the corresponding author, subject to appropriate data use agreements.***

***All PLOS journals now require all data underlying the findings described in their manuscript to be freely available to other researchers, either 1. In a public repository, 2. Within the manuscript itself, or 3. Uploaded as supplementary information.***

***This policy applies to all data except where public deposition would breach compliance with the protocol approved by your research ethics board. If your data cannot be made publicly available for ethical or legal reasons (e.g., public availability would compromise patient privacy), please explain your reasons on resubmission and your exemption request will be escalated for approval.***

***5. Please include your full ethics statement in the ‘Methods’ section of your manuscript file. In your statement, please include the full name of the IRB or ethics committee who approved or waived your study, as well as whether or not you obtained informed written or verbal consent. If consent was waived for your study, please include this information in your statement as well.***

***6. If the reviewer comments include a recommendation to cite specific previously published works, please review and evaluate these publications to determine whether they are relevant and should be cited. There is no requirement to cite these works unless the editor has indicated otherwise.***

We thank the editor for these clarifications. The Data Availability Statement has been updated to reflect that the anonymized dataset is publicly available in a stable repository (Mendeley Data; doi: 10.17632/vx7fdzkjj5.1), in accordance with PLOS ONE data sharing requirements. Accordingly, the previous statement indicating that data were available upon reasonable request has been removed.

In addition, the full ethics statement has been included in the Methods section, specifying the approving ethics committee and confirming that informed electronic consent was obtained from all participants prior to participation.

**Reviewers’ comments**

**Reviewer 1**

**General comment: Is a bit confusing who are the first authors and last authors. The most important is to mark the first Authors and or last authors.**

Thank you for this helpful comment. We have clarified authorship contributions by explicitly indicating the authors who contributed equally to the work and specifying their role. The author’s order remains unchanged, as it reflects the primary contributions to the study.

***Abstract***

***In the Results, the authors if presenting OR (CI) may decide not add p-values again. The Authors should focus on the main results.***

Thank you for this suggestion. We have revised the Results section of the abstract to improve clarity and conciseness by removing p-values and retaining effect estimates with confidence intervals.

***Methods***

***In Lines [117 – 118]. Can the Authors add the ethical approval numbers and also if the study was conducted in accordance with Helsinki declaration?***

Thank you for this important suggestion. We have added the ethical approval number and clarified that the study was conducted in accordance with the principles of the Declaration of Helsinki (Lines: 127-132).

***In Lines [130 – 132]: The Authors should consider explaining details how the Likert scales end up in scoring between 24 -120, and the subscale.***

Thank you for this suggestion. We have clarified how the Likert-scale responses are aggregated into total and subscale scores, including the number of items and scoring ranges, to improve transparency and alignment with the original instrument (Lines: 144-147).

***Line [148]: Heading and text can be merge to line [105] description of study design.***

Thank you for this suggestion. We respectfully maintained the separation between the “Study Design and Setting” and “Data Collection” sections, as they describe distinct methodological components. The former outlines the study design, setting, and timeframe, while the latter details survey administration and data handling procedures. We have, however, reviewed the text to ensure clarity and avoid any potential redundancy.

***In the Statistical Analysis, The authors should write down the statistical analyses steps in details for the main analyses.***

Thank you for this helpful suggestion. We have revised the Statistical Analysis section to describe the analytic workflow more explicitly, including the sequence of descriptive, univariate, and multivariable analyses, as well as the purpose of each model.

***Important: Instead of performing univariate, the Authors should instead for example have Table 1 with Total sample (N=xxx), non-depress (n=xxx) and depress (n=xxx). Then compute the P-values for between non-depress and depress in relation to all other variables of interest in the study (e.g, age, sex, HBI, comorbidities, etc). And present it in the results section as basic characteristics of the participants. See reference Table 1.^1^***

We thank the reviewer for this valuable suggestion. In response, we have revised Table 1 to present participant characteristics stratified by depressive symptom status (PHQ-9 <10 vs ≥10), including appropriate statistical comparisons between groups. We have also updated the Results section to highlight key differences between participants with and without clinically significant depressive symptoms, while maintaining a concise summary of the overall sample characteristics. Univariate logistic regression analyses were retained to provide effect size estimates for associations with depressive symptoms, complementing the descriptive comparisons presented in Table 1.

***The rational for using mixed-effects model should be elaborated.***

We thank the reviewer for this important comment. We have revised the Statistical Analysis section to clarify the rationale for the use of mixed-effects models. Specifically, Model A included a random intercept for academic program to account for potential clustering of participants within undergraduate and postgraduate training groups. For the subscale model, the same mixed-effects structure was initially explored; however, the model showed singularity, indicating limited variability at the random-effect level. Therefore, the final Model B was estimated using fixed-effects logistic regression (Lines: 194-206). We also clarified the statistical tests used for group comparisons in Table 1.

***Things to consider:***

***The Authors may perform the analyses in a crude model (adjusting for only sex and age) before the other model where they adjust for several confounders.***

We thank the reviewer for this helpful suggestion. We performed additional models adjusted only for age and sex to assess the stability of the associations prior to inclusion of additional covariates. These analyses showed broadly consistent results with the fully adjusted models, particularly for the association between total HBI score and depressive symptoms and the positive mental attitude subscale. Detailed results are provided in Supplementary Table 1, and this has been clarified in the Methods and Results sections (Lines: 303-306).

***The Authors should consider correcting models for multiple comparison test.***

We thank the reviewer for this important comment. The analyses conducted in this study were based on a priori hypotheses regarding the association between health behaviors and depressive symptoms, rather than exploratory testing of a large number of independent comparisons. Therefore, adjustment for multiple comparisons was not applied, in line with methodological recommendations indicating that such corrections may not be necessary in hypothesis-driven regression analyses. This approach has been clarified in the Statistical Analysis section.

***Another interesting thing to think about is, it is evidence depression is in general high among women. The Authors could perform extra sensitive analyses by performing same analyses in males and females separately.***

We thank the reviewer for this insightful suggestion. While sex-stratified analyses may provide additional insights into potential differences in associations, the present study was not specifically designed to assess effect modification by sex. Given the hypothesis-driven nature of the analyses and to maintain model parsimony, we focused on overall associations. We agree that future studies could further explore potential sex-specific differences in these relationships, and we have acknowledged this as a limitation in the Discussion section.

***Results***

***Table 2 should (HBI scores), should be merge into the new Table 1. If you presenting mean (min-max), fine and if you presenting median (IQR) fine. Don’t present both for the same variables. See Table 1 in reference 1.***

We thank the reviewer for this helpful suggestion. We have revised Table 1 to include the HBI total score as part of the baseline characteristics using consistent summary measures. In addition, we retained a separate table (Table 2) to present detailed results for HBI subscales, in order to improve clarity and avoid overloading Table 1.

***In Lines [190 – 196]: Fig. 1 can be re-worked to make more attractive and nicer, with different colors, etc. Also, either the statistical results should be in the figure (Fig. 1) or the Authors should have other table showing the statistical numbers as well.***

We thank the reviewer for this helpful suggestion. Figure 1 has been substantially revised to improve visual clarity and overall presentation. Specifically, we replaced the previous boxplot-based figure with a distribution-based visualization (raincloud plot), which better represents the underlying data by simultaneously displaying density, individual observations, and summary statistics. In addition, we have applied a clearer and more balanced color scheme to enhance readability and distinction between groups. To avoid redundancy and maintain clarity, statistical comparisons between groups are not displayed within the figure itself but are instead fully reported in Table 2, where detailed numerical results (medians, interquartile ranges, and p-values) are provided.

***In Line [205]: The idea of performing univariate analyses should be look at again. See Reference 1. Present Table 1 showing the differences between the two PHQ-9 groups. In an event you still want to do univariate perhaps correction analyses might be better.***

We thank the reviewer for this valuable suggestion. In line with this comment, Table 1 has been revised to clearly present the differences between participants with and without clinically significant depressive symptoms (PHQ-9 <10 vs ≥10), using appropriate statistical tests. Univariate logistic regression analyses were retained only as an initial step to support variable selection for multivariable models and are not presented in the Results. The main interpretation of findings is based on group comparisons (Table 1) and multivariable analyses, consistent with the analytical approach used in previous studies.

***In the Multivariate analysis. It is easier for the Authors to just write below the tables models were adjusted for xxx covariates. You don’t need to present the results with the adjusted variables. Present the main results (HBI score in Table 4 for instance was the main results). Also why does the Authors group the age variable rather than using it continuous?***

***In Table 5. Can the Authors clarify, did you include all 4-subscale of HBI in single multivariate analyses adjusting for covariates? Or it was perform on each HBI scale adjusting for covariates.***

***Perhaps, since the might cause multicollinearity, the Authors should perform them in each HBI subscale adjusting for covariates.***

***If the authors have time, they could present these main findings (without the covariates results) graphically, that would make their work attractive.***

We thank the reviewer for these insightful comments.

First, we have clarified below each table the set of covariates included in the multivariable models. While all covariates are presented to ensure transparency and allow full interpretation, we have revised the text to emphasize the main associations of interest, particularly the HBI score and its subscales.

Second, age was modeled as a categorical variable (<24 vs ≥24 years) to facilitate interpretation and reflect meaningful differences between younger and older trainees. Additionally, exploratory analyses suggested potential non-linearity in the relationship between age and depressive symptoms.

Third, in Model B, all four HBI subscales were included simultaneously in a single multivariable model together with selected covariates. This approach allows the assessment of the independent association of each subscale while accounting for the others. We assessed potential multicollinearity and did not observe evidence of instability in the estimates.

Finally, in response to the suggestion to improve visualization, Figure 1 has been revised to provide a clearer graphical representation of the distribution of HBI subscale scores according to depressive symptom status.

***Discussion***

***In Paragraph 1, the Authors should tell the study type and present the main results before discussing other findings. See reference 1.^1^***

We thank the reviewer for this helpful suggestion. The first paragraph of the Discussion has been revised to clearly state the study design and summarize the main findings prior to interpretation, in line with the recommended structure (lines: 308-313).

**Reviewer #2:**

***1. In the introduction, there is a clear statement regarding the gap in knowledge here, which aids the understanding the background of the research question. Still, the aim and specific research questions of this paper could be more clearly stated and motivated. Why do you expect PMA to be the most influential factor? Why is that important in this group in particular?***

We thank the reviewer for this insightful comment. We have revised the paragraph describing the study aim in the Introduction to more clearly articulate the rationale for focusing on Positive Mental Attitude (PMA). Specifically, we added a conceptual explanation highlighting the relevance of cognitive–emotional orientations in high-stress training environments such as medical education, and clarified why PMA was hypothesized to be the most influential behavioral domain in this population (Lines: 89-93).

***2. Regarding recruitment, how was the minimum sample estimated? Based on what research question? Is the response rate based on all responders or those who completed the questionnaire? How many were excluded due to incomplete forms? What do we know of those who were not included?***

We thank the reviewer for this important comment. We have revised the Methods section to clarify the sample size estimation and recruitment process. Specifically, we now indicate that the minimum sample size was calculated using a finite population approach based on an assumed prevalence of clinically significant depressive symptoms (PHQ-9 ≥10). We also clarified that the survey was distributed to the entire eligible population (census approach), and that the final sample exceeded the minimum required size, thereby improving the precision of the estimates (lines: 116-125).

***3. Covariate section: The analyses include all participants without subgrouping (which you mention as a limitation in the discussion). Still, night shifts was only relevant for some participants. Similarly, “paid work” appears only relevant for residences and perhaps “hours of study” is only relevant for students. You may want to consider some potential confounding effect. Especially since age was a significant predictor and likely associated with whether participants were students/residences.***

We thank the reviewer for this thoughtful observation. We acknowledge that certain covariates (e.g., night shifts, paid work, and hours of study) may have differential relevance depending on training level. In our analyses, we accounted for these potential differences by adjusting for both academic program (undergraduate vs postgraduate) and age, which are closely related to these exposures. Additionally, the hierarchical structure of the data was considered in Model A through the inclusion of a random intercept for academic program. However, for Model B, a mixed-effects specification was not retained due to singularity issues, and a fixed-effects approach was used instead. We have clarified these considerations in the Discussion as study limitations, including the absence of stratified analyses by training stage.

***4. Moreover, alcohol is not explained in Covariates section. How is that variable coded?***

We thank the reviewer for this comment. We have clarified the definition of alcohol use in the Covariates section of the Methods, specifying that it was assessed as self-reported current consumption and coded as a binary variable (yes/no) (Lines: 169-170).

***5. You could considering listing some examples of HBI questions for clarification of what is tested. An association between symptoms of depression and positive mental attitude sounds very reasonable, raising the question if the same parameter is being examined***.

We thank the reviewer for this important comment. We have added examples of items included in the Health Behavior Inventory (HBI), particularly within the Positive Mental Attitude (PMA) subscale, to improve clarity regarding the domains assessed. Additionally, we clarified the conceptual distinction between PMA and depressive symptoms measured by the PHQ-9, emphasizing that PMA reflects cognitive and behavioral strategies related to emotional regulation rather than clinical symptomatology (Lines: 147-157).

***6. Through your work, many statistical analyses are conducted, did you consider statistically adjusting for this? You may consider including a motivation otherwise.***

We thank the reviewer for this important point. Given the hypothesis-driven nature of our analyses and the pre-specification of primary exposure variables, we did not apply formal adjustments for multiple comparisons. We have clarified this rationale in the Statistical Analysis section of the Methods (Lines: 211-212).

***7. Table 4: OR is mentioned in footnote but not in the table.***

We thank the reviewer for this observation. We have corrected the footnote of Table 4 to ensure consistency with the reported values, specifying that the estimates correspond to adjusted odds ratios (AOR).

***8. Are assumptions met for statistical models?***

We thank the reviewer for this important point. We have clarified in the Statistical Analysis section that model assumptions were evaluated, including assessment of multicollinearity, model fit, and convergence, and no issues were identified that would compromise the validity of the analyses (Lines: 207-213).

***9. To aid the reader, some more explanation as to how HBI scores should be interpreted could be useful to include somewhere.***

We thank the reviewer for this helpful suggestion. We have clarified the interpretation of HBI scores in the Results section by explicitly indicating how changes in the score relate to the odds of depressive symptoms. Specifically, we now report the change in odds associated with a one-point increase in both the total HBI score and the Positive Mental Attitude (PMA) subscale, thereby improving the interpretability and clinical relevance of the findings (Lines: 280-281, 292-293).

***10. Table 3 and 4. You could consider removing the “ref” variables in the table, for brevity and clarity.***

We thank the reviewer for this helpful suggestion. We have simplified the tables by removing the reference category rows for improved clarity and brevity. Reference categories are now indicated within the comparison labels (e.g., “Female vs Male”), ensuring that the interpretation of the estimates remains explicit.

***11. In the Discussion, consider adjusting the text to better fit the main finding. There is much comment on lifestyle factors, while the main finding, and paper title, concerns positive mental attitude.***

We thank the reviewer for this valuable suggestion. We have revised the Discussion to better align with the main findings of the study. Specifically, we emphasized the role of positive mental attitude (PMA) as the only behavioral domain that remained independently associated with depressive symptoms in multivariable analyses. We clarified this point when introducing the discussion of lifestyle factors and reinforced it in the section on practical implications (Lines: 335-340).

***12. Importantly, you mention some areas of usefulness of these findings. The link between the usefulness and the findings could be made clearer. Furthermore, you recommend low-cost interventions in medical education, could you expand on whether there is evidence for suggest a recommendation?***

We thank the reviewer for this valuable comment. We have revised the Discussion to more clearly link the practical implications to the main findings of the study, particularly the independent association observed for Positive Mental Attitude (PMA). We also added a more cautious and evidence-based justification for the suggested low-cost interventions, noting that prior studies and reviews support the potential value of mindfulness-based and cognitive-behavioral approaches in improving mental health outcomes among health-professions students and trainees (Lines: 373-376).

***13. I understand that the primary aim of the study was to examine the association between health behaviours and depressive symptoms, which is addressed in the discussion. However, your findings also indicate that older males without comorbidities are less likely to experience depressive symptoms, yet this result is not explored further. This suggests that younger women with comorbidities may represent a more vulnerable group. Would it be worth highlighting and discussing this implication as well?***

We thank the reviewer for this insightful comment. We have expanded the Discussion to highlight that younger participants, females, and those with comorbidities showed higher odds of depressive symptoms, suggesting the presence of potentially more vulnerable subgroups. While subgroup-specific analyses were beyond the scope of the present study, we now emphasize the importance of targeted screening and support strategies for these groups in medical training environments (Lines: 335-340).

***14. Row 250-251, sentence could be rephrased for consistent tense, or changed from “screened” to “screen”.***

We thank the reviewer for this observation. We have revised the sentence to ensure consistency in tense and clarity. Past tense (“screened positive”) is used to describe study findings, in line with standard reporting conventions (Lines: 317-319).

***15. Row 301-302, sentence can be rephrased to be made clearer.***

We thank the reviewer for this observation. We have revised the sentence to improve clarity and ensure a more precise and readable formulation.

***16. Figure 1: titles on the x axle have been cut in the two first graphs.***

We thank the reviewer for this observation. Figure 1 has been redesigned to improve clarity and visual presentation. The updated figure now uses raincloud plots with improved layout and formatting, ensuring that all axis labels are fully visible and clearly readable.

**Reviewer 3**

***The classification of Positive Mental Attitude (PMA) as a health behavior within the HBI framework is concerning. PMA, as described, reflects cognitive-emotional orientations (optimism, resilience, cognitive reappraisal) that are more accurately characterized as psychological traits or dispositions than as behaviors. This creates a problem: PMA is conceptually adjacent to the absence of depressive symptoms, and the two constructs likely share substantial variance by definition. The authors should explicitly address this overlap and discuss whether the PMA–depression association reflects a genuine behavioral protective factor or is partly an artifact of construct overlap (i.e., high PMA scores and low PHQ-9 scores may both be indicators of the same underlying positive affect dimension).***

We thank the reviewer for this important conceptual observation. We have revised the Methods section to clarify that the Positive Mental Attitude (PMA) subscale captures cognitive–emotional orientations that may be closely related to affective states. We now explicitly acknowledge that some degree of conceptual overlap with depressive symptomatology cannot be excluded and have tempered our interpretation accordingly (Lines: 150-157).

***The direction of the PMA–depression association is entirely ambiguous: depressive symptoms are known to reduce optimism, positive affect, and cognitive reappraisal capacity (anhedonia by definition). The authors frame PMA as a potential protective factor, but the data are equally consistent with the interpretation that depression reduces PMA scores. It fundamentally limits the inferential value of the main finding. The Discussion and the Conclusion should be more cautious in its framing and explicit this conflict.***

We thank the reviewer for this important observation. We have revised both the Discussion and Conclusion to adopt a more cautious interpretation of the association between PMA and depressive symptoms. Specifically, we now explicitly acknowledge the possibility of conceptual overlap and bidirectional relationships, and avoid framing PMA as a purely protective factor (Lines: 313-315, 423-425).

***There are two multivariate models: Model A uses mixed-effects logistic regression with a random intercept for academic program; Model B uses standard fixed-effects logistic regression. No rationale is provided for this inconsistency regarding the models. If clustering by academic program was considered important enough to model in Model A, the same clustering structure should apply in Model B. The inconsistency could affect the comparability of results across models and may artificially inflate or deflate standard errors in Model B. The authors must either apply the same model structure to both, or provide a clear statistical justification for the difference.***

We thank the reviewer for this important methodological observation. We have clarified in the Statistical Analysis section that the mixed-effects structure specified for Model B resulted in singularity, indicating insufficient variability at the random-effect level to support reliable estimation. Therefore, the inclusion of a random intercept was not statistically justified, and a fixed-effects model was used to ensure model stability and valid inference. This rationale has now been explicitly stated to clarify the difference in model specification (Lines: 202-206).

***Response rates were low among students and residents, which might imply in a systematic non-response bias. Depressed participants or the ones with poorer health behavior are usually less likely to engage in research. This is not discussed***

We thank the reviewer for this important observation. We have added a statement in the limitations section acknowledging the possibility of non-response bias, noting that individuals with more severe depressive symptoms or poorer health behaviors may have been less likely to participate, potentially leading to an underestimation of the observed prevalence and associations (Lines: 392-395).

***The original HBI was developed in Polish (Juczyński, 2001; reference 21). The authors state that a "Spanish adaptation" was pilot-tested with 15 residents for linguistic clarity, but no formal psychometric validation is reported. No factor structure, no convergent validity, no test-retest reliability. It is unclear whether a validated Spanish version exists in the published literature or whether this is a de novo adaptation. Every scale, index, form.. needs to be adapted within validated standards to be considered suitable for a specific language and sometimes population***

We thank the reviewer for this important methodological observation. We agree that the HBI was originally developed in Polish and that full psychometric validation of the Spanish version was not conducted within this study. We have clarified in the Methods that the instrument was adapted and pilot-tested for linguistic clarity, and we now explicitly acknowledge in the limitations section that formal validation (e.g., factor structure, convergent validity, test–retest reliability) was not performed, which may limit the interpretability of the constructs measured (Lines: 156-157, 395-398).

***Table 1 reports "Children: No = 562 (80.29%)" and "Number of children: 0 = 566 (80.86%)."***

We thank the reviewer for identifying this inconsistency in the previous version of the manuscript. Table 1 has been revised and simplified in the current version, and the discrepancy is no longer present. The variable is now consistently reported as a binary measure (children: yes/no), ensuring internal coherence of the data presented.

***In the univariate analysis, performing night shifts was associated with reduced odds of depressive symptoms (OR = 0.66, 95% CI: 0.49–0.89, p = 0.006). This is counterintuitive given the extensive literature linking sleep disruption and shift work to mental health deterioration. A plausible explanation, or possible bias, needs to be addressed.***

We thank the reviewer for this insightful observation. We agree that the observed association between night shifts and lower odds of depressive symptoms is counterintuitive and contrasts with existing literature linking shift work to adverse mental health outcomes. We have expanded the Discussion to address this finding, noting that it may reflect residual confounding, selection effects, or contextual factors such as the normalization and expectation of night shift work within medical training environments. We emphasize that this result should be interpreted with caution (Lines: 358-364).
