# Supplementary material for: Positive mental attitude and depressive symptoms among medical students and residents: a cross-sectional study
Source: PLoS One. 2026 Jul 17;21(7):e0354032. doi: 10.1371/journal.pone.0354032 (PMC13378974; doi:10.1371/journal.pone.0354032)
Supplement: Attachment — Submitted filename: Response letter.docx [file pone.0354032.s004.docx]

**Point-by-point respond letter**

**Reviewers' comments:**

***Reviewer #2: I thank the authors for the revisions that have certainly improved the text. Here are some further suggestions for minor revisions.***

***1. Line 43: Could be clarified that it is screening above a threshold (i.e. not screening for the presence of any symptom).***

We thank the reviewer for this suggestion. We agree that the original wording could be interpreted as referring to the presence of any depressive symptom. To improve clarity, we have revised the sentence to explicitly indicate that participants screened positive for clinically significant depressive symptoms using the predefined PHQ-9 threshold (≥10). Line: 43.

***2. Lines 102-104: Is there a source that could be referenced here?***

We thank the reviewer for this suggestion. We agree that this statement would benefit from additional supporting evidence. Accordingly, we have added references documenting the association between psychological distress among medical trainees and adverse academic, clinical, and workforce-related outcomes, including impacts on professional development, intention to remain in the profession, and quality of patient care. Line: 104.

***3. Line 192: I fail to understand the reasoning behind including age and sex separately first, could you clarify?***

We thank the reviewer for this observation. The age- and sex-adjusted models were included in response to a previous reviewer’s suggestion and because age and sex are well-established correlates of both depressive symptoms and health behaviors. These intermediate models were used to assess the robustness of the observed associations before inclusion of the full set of covariates in the multivariable analyses. We have clarified this rationale in the Methods section. Lines: 191-195.

***4. Line 313: You could consider clarifying what adjustment this sentence is referring to.***

We thank the reviewer for this suggestion. We agree that the original wording could be interpreted ambiguously. We have revised the sentence to clarify that the association refers to the relationship between Positive Mental Attitude (PMA) and depressive symptoms that remained after adjustment for other HBI domains and covariates in the multivariable model. Lines: 314-317.

***5. Lines 314-316: This initial section of the Discussion is an improvement and now correctly presents a potential bi-directional relationship between PMA and depressive symptoms. However, the section is now missing a sentence on the other potential interpretation – that there may be a protective effect.***

We thank the reviewer for this insightful observation. We agree that the discussion should acknowledge both alternative interpretations of the observed association. Accordingly, we have added a sentence recognizing that, in addition to potential conceptual overlap and bidirectionality, PMA may also represent a protective factor that promotes adaptive coping and emotional resilience, thereby reducing vulnerability to depressive symptoms. Lines: 317-320.

***6. Line 317: “Consistent with these findings…” – what is this referring to?***

We thank the reviewer for this observation. We agree that the original transition was unclear, as the subsequent paragraph refers to the prevalence of clinically significant depressive symptoms rather than the preceding discussion of PMA. We have therefore revised the text to improve coherence and explicitly emphasize the high prevalence of clinically significant depressive symptoms in our sample. Lines: 322-325.

***7. Line 320: I still suggesting changing “screened” to “screen” for consistent tense – or rephrasing.***

We thank the reviewer for this suggestion. We agree that the present tense is more appropriate when referring to prevalence estimates derived from the literature. Accordingly, we have revised the sentence for consistency and clarity. Lines: 326.

***8. Line 323: consider clarifying what “elevated burden” is referring to. For example, “…of depressive symptoms amongst health care workers”.***

We thank the reviewer for this helpful suggestion. We agree that the original wording was ambiguous. The sentence has been revised to explicitly indicate that the elevated burden refers to depressive symptoms among healthcare workers in Latin America. Lines: 328-329.

***9. Lines 332-334: Is there a source you could reference here?***

We thank the reviewer for this suggestion. We agree that this statement should be supported by appropriate references. Accordingly, we have added citations documenting the impact of depressive symptoms and psychological distress on academic performance, professional development, empathy, patient care, and workforce-related outcomes among medical trainees. Line: 339.

***10. Lines 346-357: The findings and conclusions presented here are not very clearly connected to the findings of the present work. Consider revising this section of text.***

We thank the reviewer for this insightful comment. We agree that the original paragraph emphasized evidence from lifestyle intervention studies without sufficiently linking this literature to the findings of the present study. We have therefore revised the section to more explicitly connect the discussion to our results, highlighting that although overall healthy behaviors were associated with lower odds of depressive symptoms, only Positive Mental Attitude remained independently associated after adjustment. The revised text now places greater emphasis on the potential relevance of cognitive–emotional processes in medical trainees while contextualizing these findings within the broader behavioral medicine literature. Lines: 347-359.
