# Supplementary material for: Positive mental attitude and depressive symptoms among medical students and residents: a cross-sectional study
Source: PLoS One. 2026 Jul 17;21(7):e0354032. doi: 10.1371/journal.pone.0354032 (PMC13378974; doi:10.1371/journal.pone.0354032)
Supplement: Attachment — Submitted filename: Reviewer_Comments.docx [file pone.0354032.s002.docx]

Reviewer comments

In this study entitled “**Positive mental attitude and depressive symptoms among medical students and residents: a cross-sectional study.**” The authors examined the associations between health behaviors, PMA, and depressive symptoms among medical students and residents in Ecuador. The study was conducted on 700 medical students and postgraduate residents at a private university. Participants completed the Patient Health Questionnaire-9 (PHQ-9), the Health Behavior Inventory (HBI) and sociodemographic and academic information.

**General comment:** Is a bit confusing who are the first authors and last authors. The most important is to mark the first Authors and or last authors.

**Abstract**

In the **Results**, the authors if presenting OR (CI) may decide not add p-values again. The Authors should focus on the main results.

**Methods**

In Lines [117 – 118]. Can the Authors add the ethical approval numbers and also if the study was conducted in accordance with Helsinki declaration?

In Lines [130 – 132]: The Authors should consider explaining details how the Likert scales end up in scoring between 24 -120, and the subscale.

Line [148]: Heading and text can be merge to line [105] description of study design.

In the **Statistical Analysis**, The authors should write down the statistical analyses steps in details for the **main analyses**.

**Important:** Instead of performing univariate, the Authors should instead for example have Table 1 with Total sample (N=xxx), non-depress (n=xxx) and depress (n=xxx). Then compute the P-values for between non-depress and depress in relation to all other variables of interest in the study (e.g, age, sex, HBI, comorbidities, etc). And present it in the results section as basic characteristics of the participants. See reference Table 1.^1^

The rational for using mixed-effects model should be elaborated.

Things to consider:

The Authors may perform the analyses in a crude model (adjusting for only sex and age) before the other model where they adjust for several confounders.

**The Authors should consider correcting models for multiple comparison test.**

Another interesting thing to think about is, it is evidence depression is in general high among women. The Authors could perform extra sensitive analyses by performing same analyses in males and females separately.

**Results**

Table 2 should (HBI scores), should be merge into the new Table 1. If you presenting mean (min-max), fine and if you presenting median (IQR) fine. Don’t present both for the same variables. See Table 1 in reference 1.

In Lines [190 – 196]: Fig. 1 can be re-worked to make more attractive and nicer, with different colors, etc. Also, either the statistical results should be in the figure (Fig. 1) or the Authors should have other table showing the statistical numbers as well.

In Line [205]: The idea of performing univariate analyses should be look at again. See Reference 1. Present Table 1 showing the differences between the two PHQ-9 groups. In an event you still want to do univariate perhaps correction analyses might be better.

**In the Multivariate analysis**. It is easier for the Authors to just write below the tables models were adjusted for xxx covariates. You don’t need to present the results with the adjusted variables. Present the main results (HBI score in Table 4 for instance was the main results). Also why does the Authors group the age variable rather than using it continuous?

In Table 5. Can the Authors clarify, did you include all 4-subscale of HBI in single multivariate analyses adjusting for covariates? Or it was perform on each HBI scale adjusting for covariates.

Perhaps, since the might cause multicollinearity, the Authors should perform them in each HBI subscale adjusting for covariates.

If the authors have time, they could present these main findings (without the covariates results) graphically, that would make their work attractive.

**Discussion**

In **Paragraph 1**, the Authors should tell the study type and present the main results before discussing other findings. See reference 1.^1^

**References**

1.	Seo, E.H., et al., *Depressive symptoms and their associations with positive psychosocial factors among medical students.* Medicine (Baltimore), 2026. **105**(4): p. e47333.
