# Supplementary material for: Positive mental attitude and depressive symptoms among medical students and residents: a cross-sectional study
Source: PLoS One. 2026 Jul 17;21(7):e0354032. doi: 10.1371/journal.pone.0354032 (PMC13378974; doi:10.1371/journal.pone.0354032)
Supplement: Peer review history

# Peer review: Decision Letter 0

**DOI:** 10.1371/journal.pone.0354032.r001

**Authors:** Vincenzo De Luca

20 Apr 2026

PONE-D-25-69009

Positive mental attitude and depressive symptoms among medical students and residents: a cross-sectional study

PLOS One

Dear Dr. Sánchez,

Thank you for submitting your manuscript to PLOS ONE. After careful consideration, we feel that it has merit but does not fully meet PLOS ONE’s publication criteria as it currently stands. Therefore, we invite you to submit a revised version of the manuscript that addresses the points raised during the review process.

Please submit your revised manuscript by Jun 04 2026 11:59PM. If you will need more time than this to complete your revisions, please reply to this message or contact the journal office at plosone@plos.org. When you're ready to submit your revision, log on to https://www.editorialmanager.com/pone/ and select the 'Submissions Needing Revision' folder to locate your manuscript file.

Please include the following items when submitting your revised manuscript:

- A letter that responds to each point raised by the academic editor and reviewer(s). You should upload this letter as a separate file labeled 'Response to Reviewers'.
- A marked-up copy of your manuscript that highlights changes made to the original version. You should upload this as a separate file labeled 'Revised Manuscript with Track Changes'.
- An unmarked version of your revised paper without tracked changes. You should upload this as a separate file labeled 'Manuscript'.

If you would like to make changes to your financial disclosure, please include your updated statement in your cover letter. Guidelines for resubmitting your figure files are available below the reviewer comments at the end of this letter.

If applicable, we recommend that you deposit your laboratory protocols in protocols.io to enhance the reproducibility of your results. Protocols.io assigns your protocol its own identifier (DOI) so that it can be cited independently in the future. For instructions see: https://journals.plos.org/plosone/s/submission-guidelines#loc-laboratory-protocols. Additionally, PLOS ONE offers an option for publishing peer-reviewed Lab Protocol articles, which describe protocols hosted on protocols.io. Read more information on sharing protocols at https://plos.org/protocols?utm_medium=editorial-email&utm_source=authorletters&utm_campaign=protocols.

As the corresponding author, your ORCID iD is verified in the submission system and will appear in the published article. PLOS supports the use of ORCID, and we encourage all coauthors to register for an ORCID iD and use it as well. Please encourage your coauthors to verify their ORCID iD within the submission system before final acceptance, as unverified ORCID iDs will not appear in the published article. *Only*  the individual author can complete the verification step; PLOS staff *cannot*  verify ORCID iDs on behalf of authors.

We look forward to receiving your revised manuscript.

Kind regards,

Vincenzo De Luca

Academic Editor

PLOS One

Journal Requirements:

When submitting your revision, we need you to address these additional requirements.

1. Please ensure that your manuscript meets PLOS ONE's style requirements, including those for file naming. The PLOS ONE style templates can be found at

https://journals.plos.org/plosone/s/file?id=wjVg/PLOSOne_formatting_sample_main_body.pdf and

https://journals.plos.org/plosone/s/file?id=ba62/PLOSOne_formatting_sample_title_authors_affiliations.pdf

2. Please provide details regarding participant consent. In the ethics statement in the Methods and online submission information, please ensure that you have specified (1) whether consent was informed and (2) what type you obtained (for instance, written or verbal, and if verbal, how it was documented and witnessed). If your study included minors, state whether you obtained consent from parents or guardians. If the need for consent was waived by the ethics committee, please include this information.

If you are reporting a retrospective study of medical records or archived samples, please ensure that you have discussed whether all data were fully anonymized before you accessed them and/or whether the IRB or ethics committee waived the requirement for informed consent. If patients provided informed written consent to have data from their medical records used in research, please include this information.

3. We note that you have indicated that there are restrictions to data sharing for this study. For studies involving human research participant data or other sensitive data, we encourage authors to share de-identified or anonymized data. However, when data cannot be publicly shared for ethical reasons, we allow authors to make their data sets available upon request. For information on unacceptable data access restrictions, please see http://journals.plos.org/plosone/s/data-availability#loc-unacceptable-data-access-restrictions.

Before we proceed with your manuscript, please address the following prompts:

a) If there are ethical or legal restrictions on sharing a de-identified data set, please explain them in detail (e.g., data contain potentially identifying or sensitive patient information, data are owned by a third-party organization, etc.) and who has imposed them (e.g., a Research Ethics Committee or Institutional Review Board, etc.). Please also provide contact information for a data access committee, ethics committee, or other institutional body to which data requests may be sent.

b) If there are no restrictions, please upload the minimal anonymized data set necessary to replicate your study findings to a stable, public repository and provide us with the relevant URLs, DOIs, or accession numbers. Please see http://www.bmj.com/content/340/bmj.c181.long for guidelines on how to de-identify and prepare clinical data for publication. For a list of recommended repositories, please see https://journals.plos.org/plosone/s/recommended-repositories. You also have the option of uploading the data as Supporting Information files, but we would recommend depositing data directly to a data repository if possible.

Please update your Data Availability statement in the submission form accordingly.

4. In the online submission form, you indicated that de-identified data may be made available upon reasonable request to the corresponding author, subject to appropriate data use agreements.

All PLOS journals now require all data underlying the findings described in their manuscript to be freely available to other researchers, either 1. In a public repository, 2. Within the manuscript itself, or 3. Uploaded as supplementary information.

This policy applies to all data except where public deposition would breach compliance with the protocol approved by your research ethics board. If your data cannot be made publicly available for ethical or legal reasons (e.g., public availability would compromise patient privacy), please explain your reasons on resubmission and your exemption request will be escalated for approval.

5. Please include your full ethics statement in the ‘Methods’ section of your manuscript file. In your statement, please include the full name of the IRB or ethics committee who approved or waived your study, as well as whether or not you obtained informed written or verbal consent. If consent was waived for your study, please include this information in your statement as well.

6. If the reviewer comments include a recommendation to cite specific previously published works, please review and evaluate these publications to determine whether they are relevant and should be cited. There is no requirement to cite these works unless the editor has indicated otherwise.

[Note: HTML markup is below. Please do not edit.]

Reviewers' comments:

Reviewer's Responses to Questions

**Comments to the Author**

1. Is the manuscript technically sound, and do the data support the conclusions?

The manuscript must describe a technically sound piece of scientific research with data that supports the conclusions. Experiments must have been conducted rigorously, with appropriate controls, replication, and sample sizes. The conclusions must be drawn appropriately based on the data presented.

Reviewer #1: Yes

Reviewer #2: Yes

Reviewer #3: Partly

**********

2. Has the statistical analysis been performed appropriately and rigorously?

Reviewer #1: Yes

Reviewer #2: Yes

Reviewer #3: No

**********

3. Have the authors made all data underlying the findings in their manuscript fully available?

The PLOS Data policy requires authors to make all data underlying the findings described in their manuscript fully available without restriction, with rare exception (please refer to the Data Availability Statement in the manuscript PDF file). The data should be provided as part of the manuscript or its supporting information, or deposited to a public repository. For example, in addition to summary statistics, the data points behind means, medians and variance measures should be available. If there are restrictions on publicly sharing data—e.g. participant privacy or use of data from a third party—those must be specified.

Reviewer #1: Yes

Reviewer #2: No

Reviewer #3: Yes

**********

4. Is the manuscript presented in an intelligible fashion and written in standard English?

PLOS ONE does not copyedit accepted manuscripts, so the language in submitted articles must be clear, correct, and unambiguous. Any typographical or grammatical errors should be corrected at revision, so please note any specific errors here.

Reviewer #1: Yes

Reviewer #2: Yes

Reviewer #3: Yes

**********

5. Review Comments to the Author

Please use the space provided to explain your answers to the questions above. You may also include additional comments for the author, including concerns about dual publication, research ethics, or publication ethics. (Please upload your review as an attachment if it exceeds 20,000 characters)

Reviewer #1: This manuscript is interesting and relevant in advancing our knowledg in the field.

I have attached detailed comments and suggestions.

I hope it may help is strengthening your work. You may let me know if something is unclear.

Reviewer #2: This manuscript was very clear and well written. I thank the authors for their work. There are some revisions that I think would improve the quality of the paper. I list my suggestions below.

1. In the introduction, there is a clear statement regarding the gap in knowledge here, which aids the understanding the background of the research question. Still, the aim and specific research questions of this paper could be more clearly stated and motivated. Why do you expect PMA to be the most influential factor? Why is that important in this group in particular?

2. Regarding recruitment, how was the minimum sample estimated? Based on what research question? Is the response rate based on all responders or those who completed the questionnaire? How many were excluded due to incomplete forms? What do we know of those who were not included?

3. Covariate section: The analyses include all participants without subgrouping (which you mention as a limitation in the discussion). Still, night shifts was only relevant for some participants. Similarly, “paid work” appears only relevant for residences and perhaps “hours of study” is only relevant for students. You may want to consider some potential confounding effect. Especially since age was a significant predictor and likely associated with whether participants were students/residences.

4. Moreover, alcohol is not explained in Covariates section. How is that variable coded?

5. You could considering listing some examples of HBI questions for clarification of what is tested. An association between symptoms of depression and positive mental attitude sounds very reasonable, raising the question if the same parameter is being examined.

6. Through your work, many statistical analyses are conducted, did you consider statistically adjusting for this? You may consider including a motivation otherwise.

7. Table 4: OR is mentioned in footnote but not in the table.

8. Are assumptions met for statistical models?

9. To aid the reader, some more explanation as to how HBI scores should be interpreted could be useful to include somewhere.

10. Table 3 and 4. You could consider removing the “ref” variables in the table, for brevity and clarity.

11. In the Discussion, consider adjusting the text to better fit the main finding. There is much comment on lifestyle factors, while the main finding, and paper title, concerns positive mental attitude.

12. Importantly, you mention some areas of usefulness of these findings. The link between the usefulness and the findings could be made clearer. Furthermore, you recommend low-cost interventions in medical education, could you expand on whether there is evidence for suggest a recommendation?

13. I understand that the primary aim of the study was to examine the association between health behaviours and depressive symptoms, which is addressed in the discussion. However, your findings also indicate that older males without comorbidities are less likely to experience depressive symptoms, yet this result is not explored further. This suggests that younger women with comorbidities may represent a more vulnerable group. Would it be worth highlighting and discussing this implication as well?

14. Row 250-251, sentence could be rephrased for consistent tense, or changed from “screened” to “screen”.

15. Row 301-302, sentence can be rephrased to be made clearer.

16. Figure 1: titles on the x axle have been cut in the two first graphs.

Reviewer #3: The classification of Positive Mental Attitude (PMA) as a health behavior within the HBI framework is concerning. PMA, as described, reflects cognitive-emotional orientations (optimism, resilience, cognitive reappraisal) that are more accurately characterized as psychological traits or dispositions than as behaviors. This creates a problem: PMA is conceptually adjacent to the absence of depressive symptoms, and the two constructs likely share substantial variance by definition. The authors should explicitly address this overlap and discuss whether the PMA–depression association reflects a genuine behavioral protective factor or is partly an artifact of construct overlap (i.e., high PMA scores and low PHQ-9 scores may both be indicators of the same underlying positive affect dimension).

The direction of the PMA–depression association is entirely ambiguous: depressive symptoms are known to reduce optimism, positive affect, and cognitive reappraisal capacity (anhedonia by definition). The authors frame PMA as a potential protective factor, but the data are equally consistent with the interpretation that depression reduces PMA scores. It fundamentally limits the inferential value of the main finding. The Discussion and the Conclusion should be more cautious in its framing and explicit this conflict.

There are two multivariate models: Model A uses mixed-effects logistic regression with a random intercept for academic program; Model B uses standard fixed-effects logistic regression. No rationale is provided for this inconsistency regarding the models. If clustering by academic program was considered important enough to model in Model A, the same clustering structure should apply in Model B. The inconsistency could affect the comparability of results across models and may artificially inflate or deflate standard errors in Model B. The authors must either apply the same model structure to both, or provide a clear statistical justification for the difference.

Response rates were low among students and residents, which might imply in a systematic non-response bias. Depressed participants or the ones with poorer health behavior are usually less likely to engage in research. This is not discussed

The original HBI was developed in Polish (Juczyński, 2001; reference 21). The authors state that a "Spanish adaptation" was pilot-tested with 15 residents for linguistic clarity, but no formal psychometric validation is reported. No factor structure, no convergent validity, no test-retest reliability. It is unclear whether a validated Spanish version exists in the published literature or whether this is a de novo adaptation. Every scale, index, form.. needs to be adapted within validated standards to be considered suitable for a specific language and sometimes population

Table 1 reports "Children: No = 562 (80.29%)" and "Number of children: 0 = 566 (80.86%)."

In the univariate analysis, performing night shifts was associated with reduced odds of depressive symptoms (OR = 0.66, 95% CI: 0.49–0.89, p = 0.006). This is counterintuitive given the extensive literature linking sleep disruption and shift work to mental health deterioration. A plausible explanation, or possible bias, needs to be addressed.

**********

6. PLOS authors have the option to publish the peer review history of their article (what does this mean?). If published, this will include your full peer review and any attached files.

If you choose “no”, your identity will remain anonymous but your review may still be made public.

**Do you want your identity to be public for this peer review?**  For information about this choice, including consent withdrawal, please see our Privacy Policy.

Reviewer #1: **Yes:**  Nazib M Seidu

Reviewer #2: No

Reviewer #3: **Yes:**  Daniel de Siqueira Lima

**********

[NOTE: If reviewer comments were submitted as an attachment file, they will be attached to this email and accessible via the submission site. Please log into your account, locate the manuscript record, and check for the action link "View Attachments". If this link does not appear, there are no attachment files.]

To ensure your figures meet our technical requirements, please review our figure guidelines: https://journals.plos.org/plosone/s/figures

You may also use PLOS’s free figure tool, NAAS, to help you prepare publication quality figures: https://journals.plos.org/plosone/s/figures#loc-tools-for-figure-preparation.

NAAS will assess whether your figures meet our technical requirements by comparing each figure against our figure specifications.

Attachment Submitted filename: Reviewer_Comments.docx

# Author comment: Author response to Decision Letter 1

**DOI:** 10.1371/journal.pone.0354032.r002

27 Apr 2026

Point-by-point respond letter

Editor’s comments

1. Please ensure that your manuscript meets PLOS ONE's style requirements, including those for file naming. The PLOS ONE style templates can be found at

We thank the editor for this reminder. We confirm that the manuscript has been revised to comply with PLOS ONE’s style requirements, including file naming and formatting guidelines.

2. Please provide details regarding participant consent. In the ethics statement in the Methods and online submission information, please ensure that you have specified (1) whether consent was informed and (2) what type you obtained (for instance, written or verbal, and if verbal, how it was documented and witnessed). If your study included minors, state whether you obtained consent from parents or guardians. If the need for consent was waived by the ethics committee, please include this information.

We thank the editor for this clarification. We have revised the ethics statement to specify that informed consent was obtained electronically. Participants were provided with an online information sheet and indicated their consent by selecting an agreement option before accessing the survey.

If you are reporting a retrospective study of medical records or archived samples, please ensure that you have discussed whether all data were fully anonymized before you accessed them and/or whether the IRB or ethics committee waived the requirement for informed consent. If patients provided informed written consent to have data from their medical records used in research, please include this information.

We thank the editor for this clarification. This requirement is not applicable to our study, as it did not involve retrospective medical records or archived samples. Instead, data were collected prospectively through an anonymous online survey, and all participants provided informed electronic consent prior to participation.

3. We note that you have indicated that there are restrictions to data sharing for this study. For studies involving human research participant data or other sensitive data, we encourage authors to share de-identified or anonymized data. However, when data cannot be publicly shared for ethical reasons, we allow authors to make their data sets available upon request. For information on unacceptable data access restrictions, please see http://journals.plos.org/plosone/s/data-availability#loc-unacceptable-data-access-restrictions.

Before we proceed with your manuscript, please address the following prompts:

a) If there are ethical or legal restrictions on sharing a de-identified data set, please explain them in detail (e.g., data contain potentially identifying or sensitive patient information, data are owned by a third-party organization, etc.) and who has imposed them (e.g., a Research Ethics Committee or Institutional Review Board, etc.). Please also provide contact information for a data access committee, ethics committee, or other institutional body to which data requests may be sent.

b) If there are no restrictions, please upload the minimal anonymized data set necessary to replicate your study findings to a stable, public repository and provide us with the relevant URLs, DOIs, or accession numbers. Please see http://www.bmj.com/content/340/bmj.c181.long for guidelines on how to de-identify and prepare clinical data for publication. For a list of recommended repositories, please see https://journals.plos.org/plosone/s/recommended-repositories. You also have the option of uploading the data as Supporting Information files, but we would recommend depositing data directly to a data repository if possible.

Please update your Data Availability statement in the submission form accordingly.

We thank the editor for this clarification. There are no ethical or legal restrictions on data sharing. The dataset has been fully anonymized and is publicly available in a stable repository. The minimal dataset required to replicate the study findings has been deposited in Mendeley Data: https://data.mendeley.com/preview/vx7fdzkjj5. Sánchez, Xavier (2026), “Dataset on Depression Symptoms, Health Behaviors, and Academic Characteristics among Medical Students and Resident Physicians in Ecuador”, Mendeley Data, V2, doi: 10.17632/vx7fdzkjj5.2

4. In the online submission form, you indicated that de-identified data may be made available upon reasonable request to the corresponding author, subject to appropriate data use agreements.

All PLOS journals now require all data underlying the findings described in their manuscript to be freely available to other researchers, either 1. In a public repository, 2. Within the manuscript itself, or 3. Uploaded as supplementary information.

This policy applies to all data except where public deposition would breach compliance with the protocol approved by your research ethics board. If your data cannot be made publicly available for ethical or legal reasons (e.g., public availability would compromise patient privacy), please explain your reasons on resubmission and your exemption request will be escalated for approval.

5. Please include your full ethics statement in the ‘Methods’ section of your manuscript file. In your statement, please include the full name of the IRB or ethics committee who approved or waived your study, as well as whether or not you obtained informed written or verbal consent. If consent was waived for your study, please include this information in your statement as well.

6. If the reviewer comments include a recommendation to cite specific previously published works, please review and evaluate these publications to determine whether they are relevant and should be cited. There is no requirement to cite these works unless the editor has indicated otherwise.

We thank the editor for these clarifications. The Data Availability Statement has been updated to reflect that the anonymized dataset is publicly available in a stable repository (Mendeley Data; doi: 10.17632/vx7fdzkjj5.1), in accordance with PLOS ONE data sharing requirements. Accordingly, the previous statement indicating that data were available upon reasonable request has been removed.

In addition, the full ethics statement has been included in the Methods section, specifying the approving ethics committee and confirming that informed electronic consent was obtained from all participants prior to participation.

Reviewers’ comments

Reviewer 1

General comment: Is a bit confusing who are the first authors and last authors. The most important is to mark the first Authors and or last authors.

Thank you for this helpful comment. We have clarified authorship contributions by explicitly indicating the authors who contributed equally to the work and specifying their role. The author’s order remains unchanged, as it reflects the primary contributions to the study.

Abstract

In the Results, the authors if presenting OR (CI) may decide not add p-values again. The Authors should focus on the main results.

Thank you for this suggestion. We have revised the Results section of the abstract to improve clarity and conciseness by removing p-values and retaining effect estimates with confidence intervals.

Methods

In Lines [117 – 118]. Can the Authors add the ethical approval numbers and also if the study was conducted in accordance with Helsinki declaration?

Thank you for this important suggestion. We have added the ethical approval number and clarified that the study was conducted in accordance with the principles of the Declaration of Helsinki (Lines: 127-132).

In Lines [130 – 132]: The Authors should consider explaining details how the Likert scales end up in scoring between 24 -120, and the subscale.

Thank you for this suggestion. We have clarified how the Likert-scale responses are aggregated into total and subscale scores, including the number of items and scoring ranges, to improve transparency and alignment with the original instrument (Lines: 144-147).

Line [148]: Heading and text can be merge to line [105] description of study design.

Thank you for this suggestion. We respectfully maintained the separation between the “Study Design and Setting” and “Data Collection” sections, as they describe distinct methodological components. The former outlines the study design, setting, and timeframe, while the latter details survey administration and data handling procedures. We have, however, reviewed the text to ensure clarity and avoid any potential redundancy.

In the Statistical Analysis, The authors should write down the statistical analyses steps in details for the main analyses.

Thank you for this helpful suggestion. We have revised the Statistical Analysis section to describe the analytic workflow more explicitly, including the sequence of descriptive, univariate, and multivariable analyses, as well as the purpose of each model.

Important: Instead of performing univariate, the Authors should instead for example have Table 1 with Total sample (N=xxx), non-depress (n=xxx) and depress (n=xxx). Then compute the P-values for between non-depress and depress in relation to all other variables of interest in the study (e.g, age, sex, HBI, comorbidities, etc). And present it in the results section as basic characteristics of the participants. See reference Table 1.1

We thank the reviewer for this valuable suggestion. In response, we have revised Table 1 to present participant characteristics stratified by depressive symptom status (PHQ-9 <10 vs ≥10), including appropriate statistical comparisons between groups. We have also updated the Results section to highlight key differences between participants with and without clinically significant depressive symptoms, while maintaining a concise summary of the overall sample characteristics. Univariate logistic regression analyses were retained to provide effect size estimates for associations with depressive symptoms, complementing the descriptive comparisons presented in Table 1.

The rational for using mixed-effects model should be elaborated.

We thank the reviewer for this important comment. We have revised the Statistical Analysis section to clarify the rationale for the use of mixed-effects models. Specifically, Model A included a random intercept for academic program to account for potential clustering of participants within undergraduate and postgraduate training groups. For the subscale model, the same mixed-effects structure was initially explored; however, the model showed singularity, indicating limited variability at the random-effect level. Therefore, the final Model B was estimated using fixed-effects logistic regression (Lines: 194-206). We also clarified the statistical tests used for group comparisons in Table 1.

Things to consider:

The Authors may perform the analyses in a crude model (adjusting for only sex and age) before the other model where they adjust for several confounders.

We thank the reviewer for this helpful suggestion. We performed additional models adjusted only for age and sex to assess the stability of the associations prior to inclusion of additional covariates. These analyses showed broadly consistent results with the fully adjusted models, particularly for the association between total HBI score and depressive symptoms and the positive mental attitude subscale. Detailed results are provided in Supplementary Table 1, and this has been clarified in the Methods and Results sections (Lines: 303-306).

The Authors should consider correcting models for multiple comparison test.

We thank the reviewer for this important comment. The analyses conducted in this study were based on a priori hypotheses regarding the association between health behaviors and depressive symptoms, rather than exploratory testing of a large number of independent comparisons. Therefore, adjustment for multiple comparisons was not applied, in line with methodological recommendations indicating that such corrections may not be necessary in hypothesis-driven regression analyses. This approach has been clarified in the Statistical Analysis section.

Another interesting thing to think about is, it is evidence depression is in general high among women. The Authors could perform extra sensitive analyses by performing same analyses in males and females separately.

We thank the reviewer for this insightful suggestion. While sex-stratified analyses may provide additional insights into potential differences in associations, the present study was not specifically designed to assess effect modification by sex. Given the hypothesis-driven nature of the analyses and to maintain model parsimony, we focused on overall associations. We agree that future studies could further explore potential sex-specific differences in these relationships, and we have acknowledged this as a limitation in the Discussion section.

Results

Table 2 should (HBI scores), should be merge into the new Table 1. If you presenting mean (min-max), fine and if you presenting median (IQR) fine. Don’t present both for the same variables. See Table 1 in reference 1.

We thank the reviewer for this helpful suggestion. We have revised Table 1 to include the HBI total score as part of the baseline characteristics using consistent summary measures. In addition, we retained a separate table (Table 2) to present detailed results for HBI subscales, in order to improve clarity and avoid overloading Table 1.

In Lines [190 – 196]: Fig. 1 can be re-worked to make more attractive and nicer, with different colors, etc. Also, either the statistical results should be in the figure (Fig. 1) or the Authors should have other table showing the statistical numbers as well.

We thank the reviewer for this helpful suggestion. Figure 1 has been substantially revised to improve visual clarity and overall presentation. Specifically, we replaced the previous boxplot-based figure with a distribution-based visualization (raincloud plot), which better represents the underlying data by simultaneously displaying density, individual observations, and summary statistics. In addition, we have applied a clearer and more balanced color scheme to enhance readability and distinction between groups. To avoid redundancy and maintain clarity, statistical comparisons between groups are not displayed within the figure itself but are instead fully reported in Table 2, where detailed numerical results (medians, interquartile ranges, and p-values) are provided.

In Line [205]: The idea of performing univariate analyses should be look at again. See Reference 1. Present Table 1 showing the differences between the two PHQ-9 groups. In an event you still want to do univariate perhaps correction analyses might be better.

We thank the reviewer for this valuable suggestion. In line with this comment, Table 1 has been revised to clearly present the differences between participants with and without clinically significant depressive symptoms (PHQ-9 <10 vs ≥10), using appropriate statistical tests. Univariate logistic regression analyses were retained only as an initial step to support variable selection for multivariable models and are not presented in the Results. The main interpretation of findings is based on group comparisons (Table 1) and multivariable analyses, consistent with the analytical approach used in previous studies.

In the Multivariate analysis. It is easier for the Authors to just write below the tables models were adjusted for xxx covariates. You don’t need to present the results with the adjusted variables. Present the main results (HBI score in Table 4 for instance was the main results). Also why does the Authors group the age variable rather than using it continuous?

In Table 5. Can the Authors clarify, did you include all 4-subscale of HBI in single multivariate analyses adjusting for covariates? Or it was perform on each HBI scale adjusting for covariates.

Perhaps, since the might cause multicollinearity, the Authors should perform them in each HBI subscale adjusting for covariates.

If the authors have time, they could present these main findings (without the covariates results) graphically, that would make their work attractive.

We thank the reviewer for these insightful comments.

First, we have clarified below each table the set of covariates included in the multivariable models. While

Attachment Submitted filename: Response to reviewers.docx

# Peer review: Decision Letter 1

**DOI:** 10.1371/journal.pone.0354032.r003

**Authors:** Vincenzo De Luca

8 Jun 2026

PONE-D-25-69009R1

Positive mental attitude and depressive symptoms among medical students and residents: a cross-sectional study

PLOS One

Dear Dr. Sánchez,

Thank you for submitting your manuscript to PLOS ONE. After careful consideration, we feel that it has merit but does not fully meet PLOS ONE’s publication criteria as it currently stands. Therefore, we invite you to submit a revised version of the manuscript that addresses the points raised during the review process.

Please submit your revised manuscript by Jul 23 2026 11:59PM. If you will need more time than this to complete your revisions, please reply to this message or contact the journal office at plosone@plos.org. When you're ready to submit your revision, log on to https://www.editorialmanager.com/pone/ and select the 'Submissions Needing Revision' folder to locate your manuscript file.

Please include the following items when submitting your revised manuscript:

- A letter that responds to each point raised by the academic editor and reviewer(s). You should upload this letter as a separate file labeled 'Response to Reviewers'.
- A marked-up copy of your manuscript that highlights changes made to the original version. You should upload this as a separate file labeled 'Revised Manuscript with Track Changes'.
- An unmarked version of your revised paper without tracked changes. You should upload this as a separate file labeled 'Manuscript'.

If you would like to make changes to your financial disclosure, please include your updated statement in your cover letter. Guidelines for resubmitting your figure files are available below the reviewer comments at the end of this letter.

If applicable, we recommend that you deposit your laboratory protocols in protocols.io to enhance the reproducibility of your results. Protocols.io assigns your protocol its own identifier (DOI) so that it can be cited independently in the future. For instructions see: https://journals.plos.org/plosone/s/submission-guidelines#loc-laboratory-protocols. Additionally, PLOS ONE offers an option for publishing peer-reviewed Lab Protocol articles, which describe protocols hosted on protocols.io. Read more information on sharing protocols at https://plos.org/protocols?utm_medium=editorial-email&utm_source=authorletters&utm_campaign=protocols.

As the corresponding author, your ORCID iD is verified in the submission system and will appear in the published article. PLOS supports the use of ORCID, and we encourage all coauthors to register for an ORCID iD and use it as well. Please encourage your coauthors to verify their ORCID iD within the submission system before final acceptance, as unverified ORCID iDs will not appear in the published article. *Only*  the individual author can complete the verification step; PLOS staff *cannot*  verify ORCID iDs on behalf of authors.

We look forward to receiving your revised manuscript.

Kind regards,

Vincenzo De Luca

Academic Editor

PLOS One

Journal Requirements:

1. If the reviewer comments include a recommendation to cite specific previously published works, please review and evaluate these publications to determine whether they are relevant and should be cited. There is no requirement to cite these works unless the editor has indicated otherwise.

2. Please review your reference list to ensure that it is complete and correct. If you have cited papers that have been retracted, please include the rationale for doing so in the manuscript text, or remove these references and replace them with relevant current references. Any changes to the reference list should be mentioned in the rebuttal letter that accompanies your revised manuscript. If you need to cite a retracted article, indicate the article’s retracted status in the References list and also include a citation and full reference for the retraction notice.

[Note: HTML markup is below. Please do not edit.]

Reviewers' comments:

Reviewer's Responses to Questions

**Comments to the Author**

1. If the authors have adequately addressed your comments raised in a previous round of review and you feel that this manuscript is now acceptable for publication, you may indicate that here to bypass the “Comments to the Author” section, enter your conflict of interest statement in the “Confidential to Editor” section, and submit your "Accept" recommendation.

Reviewer #2: (No Response)

**********

2. Is the manuscript technically sound, and do the data support the conclusions?

The manuscript must describe a technically sound piece of scientific research with data that supports the conclusions. Experiments must have been conducted rigorously, with appropriate controls, replication, and sample sizes. The conclusions must be drawn appropriately based on the data presented.

Reviewer #2: Yes

**********

3. Has the statistical analysis been performed appropriately and rigorously?

Reviewer #2: Yes

**********

4. Have the authors made all data underlying the findings in their manuscript fully available?

The PLOS Data policy requires authors to make all data underlying the findings described in their manuscript fully available without restriction, with rare exception (please refer to the Data Availability Statement in the manuscript PDF file). The data should be provided as part of the manuscript or its supporting information, or deposited to a public repository. For example, in addition to summary statistics, the data points behind means, medians and variance measures should be available. If there are restrictions on publicly sharing data—e.g. participant privacy or use of data from a third party—those must be specified.

Reviewer #2: Yes

**********

5. Is the manuscript presented in an intelligible fashion and written in standard English?

PLOS ONE does not copyedit accepted manuscripts, so the language in submitted articles must be clear, correct, and unambiguous. Any typographical or grammatical errors should be corrected at revision, so please note any specific errors here.

Reviewer #2: Yes

**********

6. Review Comments to the Author

Please use the space provided to explain your answers to the questions above. You may also include additional comments for the author, including concerns about dual publication, research ethics, or publication ethics. (Please upload your review as an attachment if it exceeds 20,000 characters)

Reviewer #2: I thank the authors for the revisions that have certainly improved the text. Here are some further suggestions for minor revisions.

1. Line 43: Could be clarified that it is screening above a threshold (i.e. not screening for the presence of any symptom).

2. Lines 102-104: Is there a source that could be referenced here?

3. Line 192: I fail to understand the reasoning behind including age and sex separately first, could you clarify?

4. Line 313: You could consider clarifying what adjustment this sentence is referring to.

5. Lines 314-316: This initial section of the Discussion is an improvement and now correctly presents a potential bi-directional relationship between PMA and depressive symptoms. However, the section is now missing a sentence on the other potential interpretation – that there may be a protective effect.

6. Line 317: “Consistent with these findings…” – what is this referring to?

7. Line 320: I still suggesting changing “screened” to “screen” for consistent tense – or rephrasing.

8. Line 323: consider clarifying what “elevated burden” is referring to. For example, “…of depressive symptoms amongst health care workers”.

9. Lines 332-334: Is there a source you could reference here?

10. Lines 346-357: The findings and conclusions presented here are not very clearly connected to the findings of the present work. Consider revising this section of text.

**********

7. PLOS authors have the option to publish the peer review history of their article (what does this mean?). If published, this will include your full peer review and any attached files.

If you choose “no”, your identity will remain anonymous but your review may still be made public.

**Do you want your identity to be public for this peer review?**  For information about this choice, including consent withdrawal, please see our Privacy Policy.

Reviewer #2: No

**********

[NOTE: If reviewer comments were submitted as an attachment file, they will be attached to this email and accessible via the submission site. Please log into your account, locate the manuscript record, and check for the action link "View Attachments". If this link does not appear, there are no attachment files.]

To ensure your figures meet our technical requirements, please review our figure guidelines: https://journals.plos.org/plosone/s/figures

You may also use PLOS’s free figure tool, NAAS, to help you prepare publication quality figures: https://journals.plos.org/plosone/s/figures#loc-tools-for-figure-preparation.

NAAS will assess whether your figures meet our technical requirements by comparing each figure against our figure specifications.

# Author comment: Author response to Decision Letter 2

**DOI:** 10.1371/journal.pone.0354032.r004

11 Jun 2026

Reviewers' comments:

Reviewer #2: I thank the authors for the revisions that have certainly improved the text. Here are some further suggestions for minor revisions.

1. Line 43: Could be clarified that it is screening above a threshold (i.e. not screening for the presence of any symptom).

We thank the reviewer for this suggestion.

We agree that the original wording could be interpreted as referring to the presence of any depressive symptom. To improve clarity, we have revised the sentence to explicitly indicate that participants screened positive for clinically significant depressive symptoms using the predefined PHQ-9 threshold (≥10). Line: 43.

2. Lines 102-104: Is there a source that could be referenced here?

We thank the reviewer for this suggestion. We agree that this statement would benefit from additional supporting evidence. Accordingly, we have added references documenting the association between psychological distress among medical trainees and adverse academic, clinical, and workforce-related outcomes, including impacts on professional development, intention to remain in the profession, and quality of patient care. Line: 104.

3. Line 192: I fail to understand the reasoning behind including age and sex separately first, could you clarify?

We thank the reviewer for this observation. The age- and sex-adjusted models were included in response to a previous reviewer’s suggestion and because age and sex are well-established correlates of both depressive symptoms and health behaviors. These intermediate models were used to assess the robustness of the observed associations before inclusion of the full set of covariates in the multivariable analyses. We have clarified this rationale in the Methods section. Lines: 191-195.

4. Line 313: You could consider clarifying what adjustment this sentence is referring to.

We thank the reviewer for this suggestion. We agree that the original wording could be interpreted ambiguously. We have revised the sentence to clarify that the association refers to the relationship between Positive Mental Attitude (PMA) and depressive symptoms that remained after adjustment for other HBI domains and covariates in the multivariable model. Lines: 314-317.

5. Lines 314-316: This initial section of the Discussion is an improvement and now correctly presents a potential bi-directional relationship between PMA and depressive symptoms. However, the section is now missing a sentence on the other potential interpretation – that there may be a protective effect.

We thank the reviewer for this insightful observation. We agree that the discussion should acknowledge both alternative interpretations of the observed association. Accordingly, we have added a sentence recognizing that, in addition to potential conceptual overlap and bidirectionality, PMA may also represent a protective factor that promotes adaptive coping and emotional resilience, thereby reducing vulnerability to depressive symptoms. Lines: 317-320.

6. Line 317: “Consistent with these findings…” – what is this referring to?

We thank the reviewer for this observation. We agree that the original transition was unclear, as the subsequent paragraph refers to the prevalence of clinically significant depressive symptoms rather than the preceding discussion of PMA. We have therefore revised the text to improve coherence and explicitly emphasize the high prevalence of clinically significant depressive symptoms in our sample. Lines: 322-325.

7. Line 320: I still suggesting changing “screened” to “screen” for consistent tense – or rephrasing.

We thank the reviewer for this suggestion. We agree that the present tense is more appropriate when referring to prevalence estimates derived from the literature. Accordingly, we have revised the sentence for consistency and clarity. Lines: 326.

8. Line 323: consider clarifying what “elevated burden” is referring to. For example, “…of depressive symptoms amongst health care workers”.

We thank the reviewer for this helpful suggestion. We agree that the original wording was ambiguous. The sentence has been revised to explicitly indicate that the elevated burden refers to depressive symptoms among healthcare workers in Latin America. Lines: 328-329.

9. Lines 332-334: Is there a source you could reference here?

We thank the reviewer for this suggestion. We agree that this statement should be supported by appropriate references. Accordingly, we have added citations documenting the impact of depressive symptoms and psychological distress on academic performance, professional development, empathy, patient care, and workforce-related outcomes among medical trainees. Line: 339.

10. Lines 346-357: The findings and conclusions presented here are not very clearly connected to the findings of the present work. Consider revising this section of text.

We thank the reviewer for this insightful comment. We agree that the original paragraph emphasized evidence from lifestyle intervention studies without sufficiently linking this literature to the findings of the present study. We have therefore revised the section to more explicitly connect the discussion to our results, highlighting that although overall healthy behaviors were associated with lower odds of depressive symptoms, only Positive Mental Attitude remained independently associated after adjustment. The revised text now places greater emphasis on the potential relevance of cognitive–emotional processes in medical trainees while contextualizing these findings within the broader behavioral medicine literature. Lines: 347-359.

Attachment Submitted filename: Response letter.docx

# Peer review: Decision Letter 2

**DOI:** 10.1371/journal.pone.0354032.r005

**Authors:** Vincenzo De Luca

2 Jul 2026

Positive mental attitude and depressive symptoms among medical students and residents: a cross-sectional study

PONE-D-25-69009R2

Dear Dr. Sánchez,

We’re pleased to inform you that your manuscript has been judged scientifically suitable for publication and will be formally accepted for publication once it meets all outstanding technical requirements.

Within one week, you’ll receive an e-mail detailing the required amendments. When these have been addressed, you’ll receive a formal acceptance letter and your manuscript will be scheduled for publication.

An invoice will be generated when your article is formally accepted. Please note, if your institution has a publishing partnership with PLOS and your article meets the relevant criteria, all or part of your publication costs will be covered. Please make sure your user information is up-to-date by logging into Editorial Manager at Editorial Manager® and clicking the ‘Update My Information' link at the top of the page. For questions related to billing, please contact billing support.

If your institution or institutions have a press office, please notify them about your upcoming paper to help maximize its impact. If they’ll be preparing press materials, please inform our press team as soon as possible -- no later than 48 hours after receiving the formal acceptance. Your manuscript will remain under strict press embargo until 2 pm Eastern Time on the date of publication. For more information, please contact onepress@plos.org.

Kind regards,

Vincenzo De Luca

Academic Editor

PLOS One

Additional Editor Comments (optional):

Reviewers' comments:

Reviewer's Responses to Questions

**Comments to the Author**

1. If the authors have adequately addressed your comments raised in a previous round of review and you feel that this manuscript is now acceptable for publication, you may indicate that here to bypass the “Comments to the Author” section, enter your conflict of interest statement in the “Confidential to Editor” section, and submit your "Accept" recommendation.

Reviewer #2: All comments have been addressed

**********

2. Is the manuscript technically sound, and do the data support the conclusions?

The manuscript must describe a technically sound piece of scientific research with data that supports the conclusions. Experiments must have been conducted rigorously, with appropriate controls, replication, and sample sizes. The conclusions must be drawn appropriately based on the data presented.

Reviewer #2: Yes

**********

3. Has the statistical analysis been performed appropriately and rigorously?

Reviewer #2: Yes

**********

4. Have the authors made all data underlying the findings in their manuscript fully available?

The PLOS Data policy requires authors to make all data underlying the findings described in their manuscript fully available without restriction, with rare exception (please refer to the Data Availability Statement in the manuscript PDF file). The data should be provided as part of the manuscript or its supporting information, or deposited to a public repository. For example, in addition to summary statistics, the data points behind means, medians and variance measures should be available. If there are restrictions on publicly sharing data—e.g. participant privacy or use of data from a third party—those must be specified.

Reviewer #2: Yes

**********

5. Is the manuscript presented in an intelligible fashion and written in standard English?

PLOS ONE does not copyedit accepted manuscripts, so the language in submitted articles must be clear, correct, and unambiguous. Any typographical or grammatical errors should be corrected at revision, so please note any specific errors here.

Reviewer #2: Yes

**********

6. Review Comments to the Author

Please use the space provided to explain your answers to the questions above. You may also include additional comments for the author, including concerns about dual publication, research ethics, or publication ethics. (Please upload your review as an attachment if it exceeds 20,000 characters)

Reviewer #2: Thank you for these revisions. One detail: Line 43: I think most guidelines suggest putting "(...;...)" rather than two parentheses next to each other.

**********

7. PLOS authors have the option to publish the peer review history of their article (what does this mean?). If published, this will include your full peer review and any attached files.

If you choose “no”, your identity will remain anonymous but your review may still be made public.

**Do you want your identity to be public for this peer review?**  For information about this choice, including consent withdrawal, please see our Privacy Policy.

Reviewer #2: No

**********

# Editor report: Acceptance letter

**DOI:** 10.1371/journal.pone.0354032.r006

**Authors:** Vincenzo De Luca

PONE-D-25-69009R2

PLOS One

Dear Dr. Sánchez,

I'm pleased to inform you that your manuscript has been deemed suitable for publication in PLOS One. Congratulations! Your manuscript is now being handed over to our production team.

At this stage, our production department will prepare your paper for publication. This includes ensuring the following:

* All references, tables, and figures are properly cited

* All relevant supporting information is included in the manuscript submission,

* There are no issues that prevent the paper from being properly typeset

You will receive further instructions from the production team, including instructions on how to review your proof when it is ready. Please keep in mind that we are working through a large volume of accepted articles, so please give us a few days to review your paper and let you know the next and final steps.

Lastly, if your institution or institutions have a press office, please let them know about your upcoming paper now to help maximize its impact. If they'll be preparing press materials, please inform our press team within the next 48 hours. Your manuscript will remain under strict press embargo until 2 pm Eastern Time on the date of publication. For more information, please contact onepress@plos.org.

You will receive an invoice from PLOS for your publication fee after your manuscript has reached the completed accept phase. If you receive an email requesting payment before acceptance or for any other service, this may be a phishing scheme. Learn how to identify phishing emails and protect your accounts at https://explore.plos.org/phishing.

If we can help with anything else, please email us at customercare@plos.org.

Thank you for submitting your work to PLOS ONE and supporting open access.

Kind regards,

PLOS ONE Editorial Office Staff

on behalf of

Dr. Vincenzo De Luca

Academic Editor

PLOS One
